# Forensic document examination and algorithmic handwriting analysis of Judahite biblical period inscriptions reveal significant literacy level

**DOI:** 10.1371/journal.pone.0237962

**Published:** 2020-09-09

**Authors:** Arie Shaus, Yana Gerber, Shira Faigenbaum-Golovin, Barak Sober, Eli Piasetzky, Israel Finkelstein

**Affiliations:** 1 Department of Applied Mathematics, Tel Aviv University, Tel Aviv, Israel; 2 Jacob M. Alkow Department of Archaeology and Ancient Near Eastern Civilizations, Tel Aviv University, Tel Aviv, Israel; 3 Department of Genetics, Harvard Medical School, Boston, MA, United States of America; 4 Division of Identification & Forensic Science, Retired Senior Questioned Document Examiner, Israel Police, Tel Aviv, Israel; 5 Department of Mathematics, Duke University, Durham, NC, United States of America; 6 Rhodes Information Initiative, Duke University, Durham, NC, United States of America; 7 School of Physics and Astronomy, Tel Aviv University, Tel Aviv, Israel; University of California Los Angeles, UNITED STATES

## Abstract

Arad is a well preserved desert fort on the southern frontier of the biblical kingdom of Judah. Excavation of the site yielded over 100 Hebrew ostraca (ink inscriptions on potsherds) dated to ca. 600 BCE, the eve of Nebuchadnezzar’s destruction of Jerusalem. Due to the site’s isolation, small size and texts that were written in a short time span, the Arad corpus holds important keys to understanding dissemination of literacy in Judah. Here we present the handwriting analysis of 18 Arad inscriptions, including more than 150 pair-wise assessments of writer’s identity. The examination was performed by two new algorithmic handwriting analysis methods and independently by a professional forensic document examiner. To the best of our knowledge, no such large-scale pair-wise assessments of ancient documents by a forensic expert has previously been published. Comparison of forensic examination with algorithmic analysis is also unique. Our study demonstrates substantial agreement between the results of these independent methods of investigation. Remarkably, the forensic examination reveals a high probability of at least 12 writers within the analyzed corpus. This is a major increment over the previously published algorithmic estimations, which revealed 4–7 writers for the same assemblage. The high literacy rate detected within the small Arad stronghold, estimated (using broadly-accepted paleo-demographic coefficients) to have accommodated 20–30 soldiers, demonstrates widespread literacy in the late 7^th^ century BCE Judahite military and administration apparatuses, with the ability to compose biblical texts during this period a possible by-product.

## Introduction

**Fig 1:**
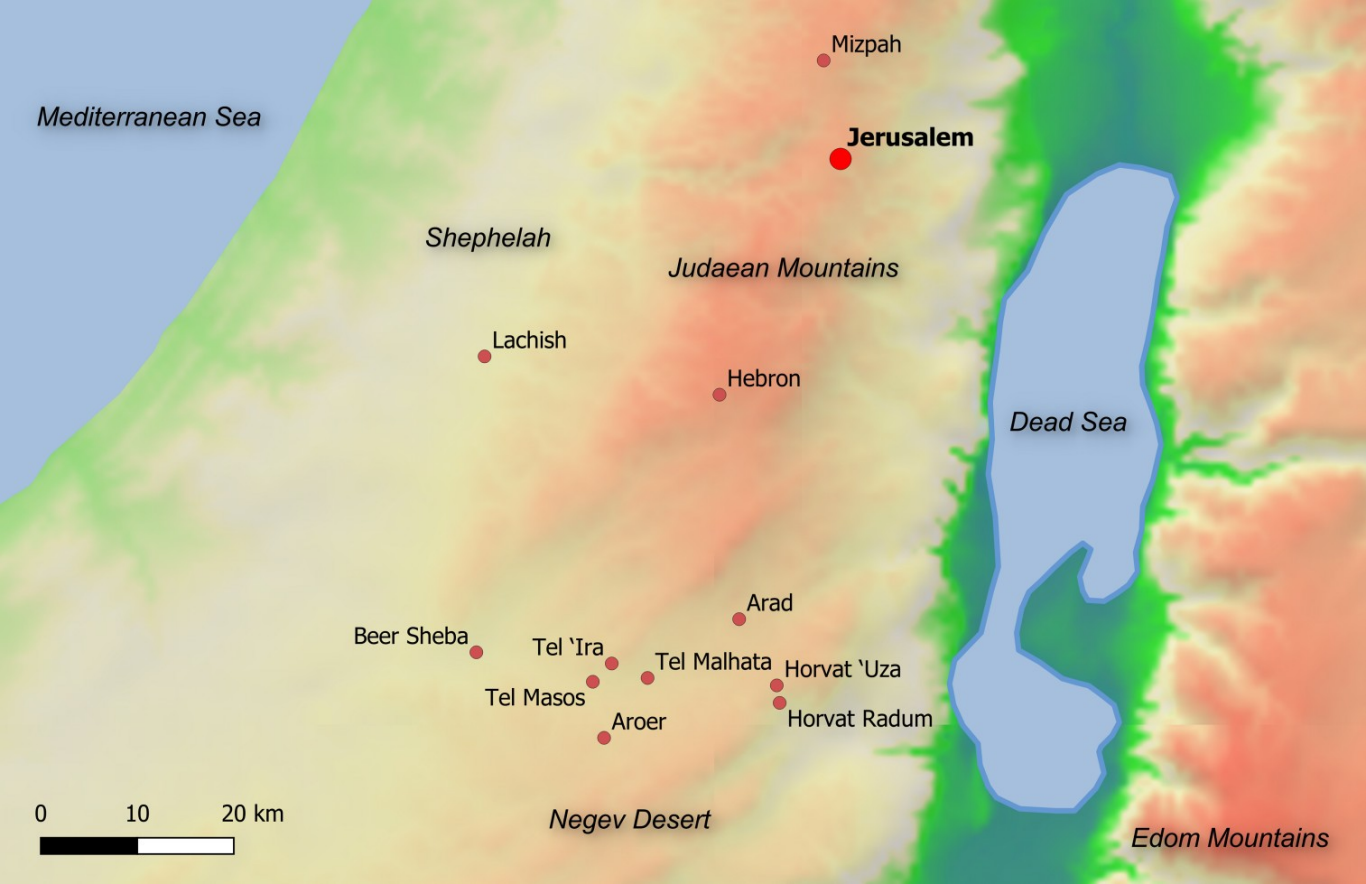
Main towns in Judah and sites in the Beer Sheba valley ca. 600 BCE.

**Fig 2:**
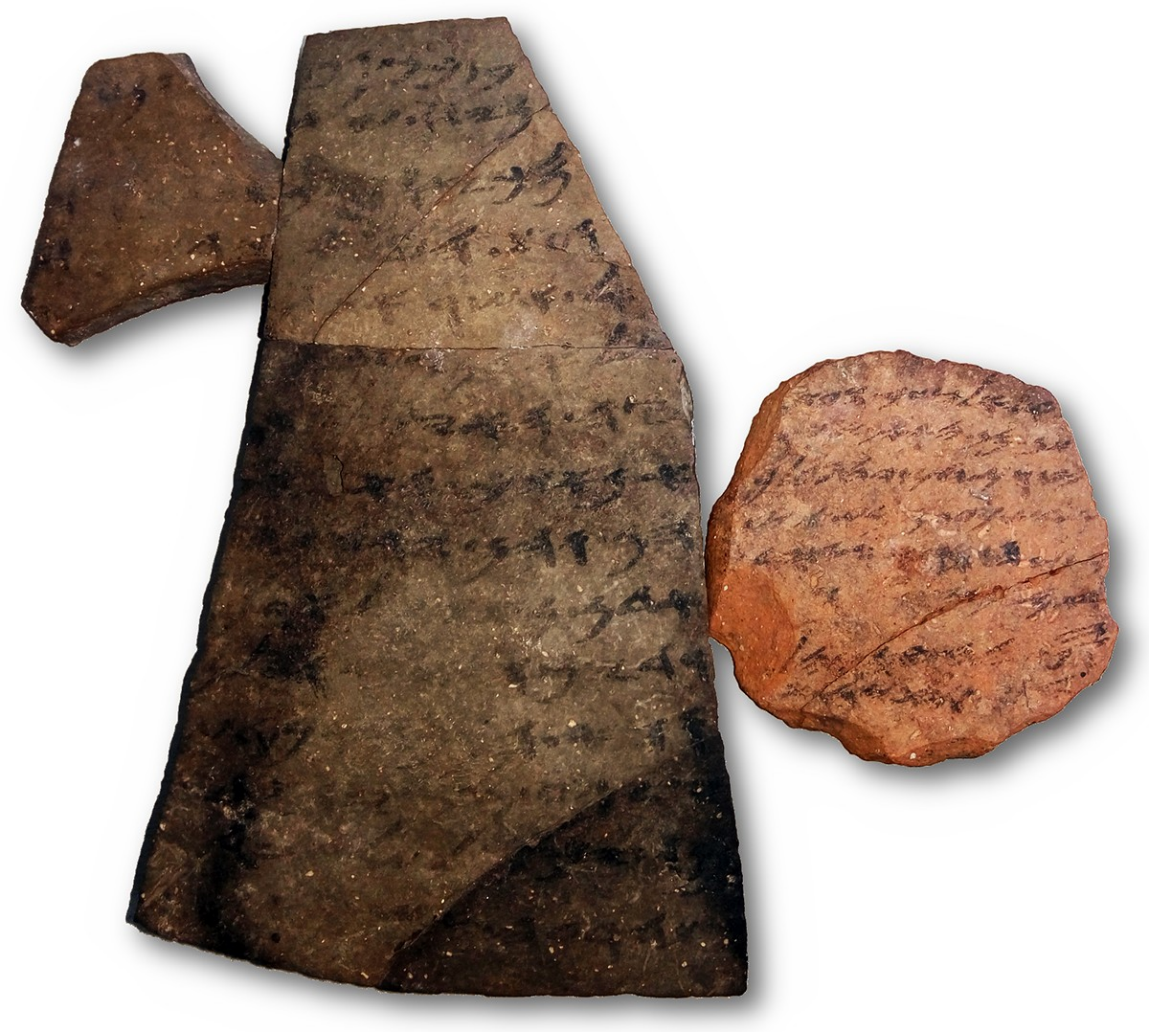
Examples of two Hebrew ostraca from Arad. Left: Ostracon 40 (9.5x14.6 cm), right: Ostracon 3 (6.0x5.9 cm). The poor state of preservation, including stains, cracks and blurred text, is apparent. The clay sherds are significantly different in shape, size, type of clay, and in their handwriting. Image courtesy of Yana Gerber and the Israel Antiquities Authority.

The Hebrew inscriptions from the Arad fort [‎1], located in the arid southern frontier of biblical Judah (see Fig 1), is one of a few textual corpora from the First Temple period. Dated to ca. 600 BCE, the more than 100 ostraca (texts written in ink on clay potsherds) provide a record of distribution of provisions to military units shortly before the destruction of the Kingdom of Judah by the invading Babylonian army (examples of some Arad ostraca are shown in Fig 2; the ostraca numbers used throughout this work are according to [1]).

The texts include administrative records, such as lists of names, probably produced at the fort itself, as well as orders that were dispatched to Arad from higher echelons in the Judahite military system, as well as correspondence with neighboring forts. One of the inscriptions mentions “the King of Judah” and another “the house of YHWH,” probably referring to the Temple in Jerusalem. Some orders of provisions refer to the *Kittiyim*, seemingly a Greek mercenary unit/s [2], which assisted in protecting the Negev desert border from the neighboring Kingdom of Edom (see Fig 1). A vital part of the corpus, the so-called “Eliashib's letters,” involving the fort quartermaster, probably encompasses the registration of about one month's expenses [3]. This is true at least for texts 1, 2, 3, 5, 7, 8, 16, 17a, 17b and 18 analyzed herein. Since, arguably, some other ostraca mention the same individuals (e.g., a son of Eliashib is mentioned within a list of names in Ostracon 38; Malkiyahu, probably the commander of the fortress, is mentioned in Ostraca 24 and 40), in our view, the same short life span is true for the majority of, or even the entire corpus. The texts provide invaluable information regarding daily life of the Judahite army personnel (e.g., [4,5]), and contribute to the research fields of history of Ancient Israel, Hebrew epigraphy and biblical exegesis.

In our previous studies [6–8] we touched upon the important topic of the literacy level in late-monarchic (7^th^ century BCE) Judah, which has ramifications for the question of composition of biblical texts during this period [9]. Due to its remote location, small size (it accommodated only ca. 20–30 soldiers) and its rich collection of texts, probably written within a short period of time [3], Arad is an excellent testing ground for examining this issue [10].

Our algorithmic studies aimed, therefore, at identifying the number of “hands” (distinct writers) in the Arad corpus [6–8]. These encompassed 16 Arad ostraca, but since two of them were double-sided, the number of texts analyzed was, in fact, 18. The same sample set is analyzed below (note that at the time the current study was conducted, we still did not know that yet another ostracon from this set was double-sided [4,5]). Our algorithmic investigations estimated a minimal number of 4, 5 or 7 writers at Arad [6–8], possibly hinting at the existence of a Judahite educational system that trained personnel for the Judahite administration, including the military (see examples of non-military writing in [11–14]).

Forensic handwriting examination of the Arad inscriptions has never before been conducted. In fact, to the best of our knowledge, such an examination has never been performed on any ancient inscription (though, see [15] for forensic chemical analysis in the context of historical texts). All the more so, the introduction of sophisticated computer-assisted forensic examination methods [16–19] (especially in the field of computerized signature forgery detection [20,21]) did not lead to combined forensic/algorithmic efforts related to historical texts.

A related, active domain of research is computerized writer identification (both modern and historical), which does not involve the expertise of professional forensic document examiners. Instead, within the context of historical texts, computerized writer identification relies on annotation of epigraphers or paleographers–specialists on ancient writing systems. Examples of such studies cover topics as diverse as ancient Greek inscriptions [22]; Byzantine and Spanish Medieval codices [23,24]; Herman Melville’s alleged 19^th^ c. texts [25]; 13^th^–20^th^ c. Arabic and Turkish manuscripts [26–28]; as well as Hebrew Second Temple [29] and Medieval [30,31] documents. This means that an often subjective opinion of “manuscript historians” (borrowing a term from [25]) is preferred over advice from professional forensic writing experts. For an in-depth analysis and comparison of the two differing methodologies see [32].

A review of [22–31] and other computerized writer identification surveys [33,34] reveals another potential problem. Commonly, the employed algorithms utilize computer vision and machine learning features and procedures to produce abstract “distances” between inscriptions–e.g., based on the slant of their characters, their relative proportions, their uniformity, etc. Such an approach only allows identifying “close” texts; it does not evaluate the quality of the match statistically. For example, if a distance between texts *A* and *B* is, say, 3.14, the chances of the writers being identical could be 1%, 10%, or 99%. The distance in itself does not convey any probabilistic information, and is insufficient for identicalness/distinctiveness ruling.

It is thus not surprising that the performance tests of such writer identification algorithms are often weak, and they are typically applied in tightly engineered environments. A “ground truth,” i.e., a collection of texts with pre-established writers, is required. The testing is not performed by comparing all possible pairs of documents within the collection. Instead, for each given document *X*, *k* “closest” inscriptions are selected, and a “success” is marked if *one* of their writers is the same as in *X*. Such distance-measuring or testing procedures are entirely irrelevant outside of a ground-truth framework. It is certainly insufficient for the task of analyzing afresh a corpus of many inscriptions with unknown writers’ identities.

Alternative algorithmic frameworks, obtaining significance levels (p-value) for writer’s identicalness of two given inscriptions, have recently been proposed [6–8]. These techniques do not require ground truth, even when operating on an entirely new, possibly ancient, collection of texts. The algorithmic methods elaborated upon in the current paper are major developments of some of these schemes [6,7].

The main contributions of this article are:

- A first of its kind detailed forensic handwriting examination of ancient inscriptions, performed on the Arad corpus.
- Two enhanced writer identification algorithms, also tested on the Arad ostraca.
- A systematic comparison between the forensic and the algorithmic results.
- Progress on the question of number of writers at Arad, with the ability to compose/copy biblical-genre texts in Judah during this period a possible by-product.

## Materials and methods

Herein we provide a brief description of the datasets, the workflow of the document examiner and the two algorithms employed. Additional details are provided in the S1 File. Throughout the article, by “character” we denote a particular instance of a given letter (e.g., there may be many characters, which are all occurrences of the letter *alep*).

### Datasets

The study was conducted on two datasets of written material. The main assemblage was a corpus of 16 Hebrew ostraca found at Arad. The inscriptions were composed during the span of a few years, ca. 600 BCE, and consist mainly of military correspondence [1]. The computerized research was performed on digital images of these inscriptions. The texts under examination were Arad Ostraca 1, 2, 3, 5, 7, 8, 16, 17, 18, 21, 24, 31, 38, 39, 40 and 111, chosen because of their relative clarity and potential for character reconstruction. Ostraca 17 and 39 contain substantial writing on both sides of the potsherd and were treated as separate texts (17a and 17b; 39a and 39b), resulting in 18 texts under examination. During the time when the research was conducted, we had not yet obtained the data from the newly discovered (via multispectral imaging) *verso* side of Arad Ostracon 16 [4,5]; thus, we have used only its *recto* in the current examination. For the forensic handwriting analysis, either the ostraca themselves, or their high quality regular or multispectral images [4,5,35–37] were used; other promising techniques of image acquisition [38,39] were less fruitful. For the algorithmic analysis, a semi-automatic reconstruction of the most prominent characters was utilized [40]; it can be downloaded at [41].

Permits for imaging, research and publication of the Arad ostraca were obtained from the Israel Antiquities Authority; see Table 1 for ostraca details. All necessary permits were obtained for the described study, which complied with all relevant regulations.

**Table 1:** Arad Hebrew ostraca details.

Ostraca No.	Israel Antiquities Authority registration No.	Current Location	Length (cm)	Width (cm)	Notes
1	1967–713	The Israel Museum, Jerusalem	8.3	5.1	
2	1967–625	The Israel Museum, Jerusalem	9.9	7.5	
3	1967–623	The Israel Museum, Jerusalem	6.0	5.9	
5	1967–627	The Israel Museum, Jerusalem	5.2	2.7	
7	1972–165	The Israel Museum, Jerusalem	7.8	5.0	
8	1967–1893	Israel Antiquities Authority, Beit Shemesh storage facility	5.6	6.6	
16	1967–990	The Israel Museum, Jerusalem	9.0	6.4	Only the *recto* was analyzed
17	1967–624	The Israel Museum, Jerusalem	8.4	6.0	*Recto* and *verso* were analyzed separately
18	1967–669	The Israel Museum, Jerusalem	6.6	4.2	
21	1972–126	Eretz Israel Museum, Tel Aviv	11.5	12.0	
24	1972–121	The Israel Museum, Jerusalem	10.5	10.0	
31	1967–1223	On loan	16.0	16.0	
38	1967–1878	Israel Antiquities Authority, Beit Shemesh storage facility	4.5	4.4	
39	1967–992	The Israel Museum, Jerusalem	5.6	5.9	*Recto* and *verso* were analyzed separately
40	1967–631	The Israel Museum, Jerusalem	14.6	9.5	
111	1967–2263	Israel Antiquities Authority, Beit Shemesh storage facility	9.3	5.4	

The second dataset, used to validate the two algorithms, contained handwriting samples collected from 18 present-day writers of modern Hebrew. This dataset allowed us to estimate the False Positive and False Negative rates for the algorithmic methods that we employed; it can be downloaded at [42]. It will be stressed that the modern Hebrew dataset was not used to train or calibrate the algorithm for its activation on the first, ancient Hebrew dataset (or vice versa). The purposes of the modern Hebrew dataset were algorithm verification and sanity check.

For additional details regarding the datasets, see S1 File, Section 1.

### Forensic handwriting examination workflow

Modern forensic handwriting examination relies on the fact that the task of writing requires the individual to combine sensory-motor skills with certain personal inclinations. Thus, it can serve as a unique identifier for the person performing the act of writing (i.e., a biometric “fingerprint”) [43]. Forensic handwriting analysis aims at tracking features corresponding to specific individuals, and utilizing them to decide whether the observed documents were written by a single hand or by different writers [44–49]. The procedure detailed below follows the protocol of modern forensic handwriting examination, adapted to ancient ostraca, utilizing many common characteristics of ancient and modern Hebrew writing (e.g., basically the same language; same alphabet; mostly separated characters; etc.)

**Fig 3:**
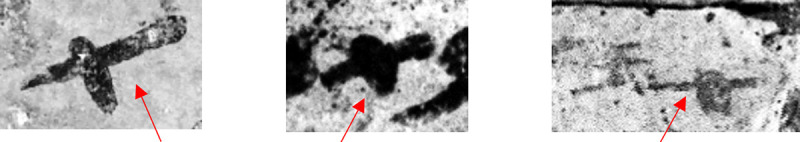
Examples of different shapes, slants, relative length, width and intersection points of the horizontal and vertical shaft of the letter *taw*. Left: Ostracon 7, middle: Ostracon 1, right: Ostracon 24.

The examination process is divided into three steps: *analysis*, *comparison*, and *evaluation*. The *analysis* phase includes a detailed examination of every single inscription and, if necessary, its high quality regular or multispectral images [4‎,5,35–37], according to the following features (for an example, see Fig 3):

- **General appearance of the sherd**: size, form and type of pottery.
- **Writing style**: legibility, writing skill and flow, and line quality.
- **Arrangement and use of space**: margins, spacing, alignment and formatting.
- **Size and proportions**: absolute and relative size of the writing and letters, alterations of size or height of upstrokes and downstrokes.
- **Slant**: general slant of the writing as well as an absolute and relative slant of letters.
- **Punctuation**: presence, form and position relative to the imaginary baseline of punctuation marks (or upper line in the case of Hebrew script).
- **Spacing**: spacing between letters, strokes, words and lines; relative position of letters vis-à-vis the preceding and following ones.
- **Alignment**: alignment of words and letters relative to an imaginary baseline.
- **Letter shapes and range of their variations within a script**: extraction of distinctive features.

The next phase of the examination process is *comparison* of writing features in different ostraca based on the aforementioned analysis. Consistent patterns and repetitions, characteristic to various inscriptions, are identified. Finally, an *evaluation* of identicalness or distinctiveness of various writers is made, using the scales of conclusions common in the forensic handwriting analysis. The grades range from the definite conclusion of identity to the definite elimination of identity [50,51]. Inconclusive grade is used when there are significant limiting factors in the investigated or in known handwriting, and the examiner does not lean in one direction or another.

For additional details regarding the forensic handwriting analysis procedure, see S1 File, Section 2.

### Algorithm #1: Writers’ identification via a combination of features

The algorithm aims at differentiating between writers of a given set of texts. The method described below is an improvement and enhancement of an algorithm previously published in [6]. The main alterations are: replacement of k-mean clustering with k-medoid clustering; improved representation of non-homogeneity of the characters; updated and more accurate p-value calculation; lowering the p-value threshold to 0.l for significance-enhancing purposes (for further details, see S1 File, Section 3).

**Fig 4:**
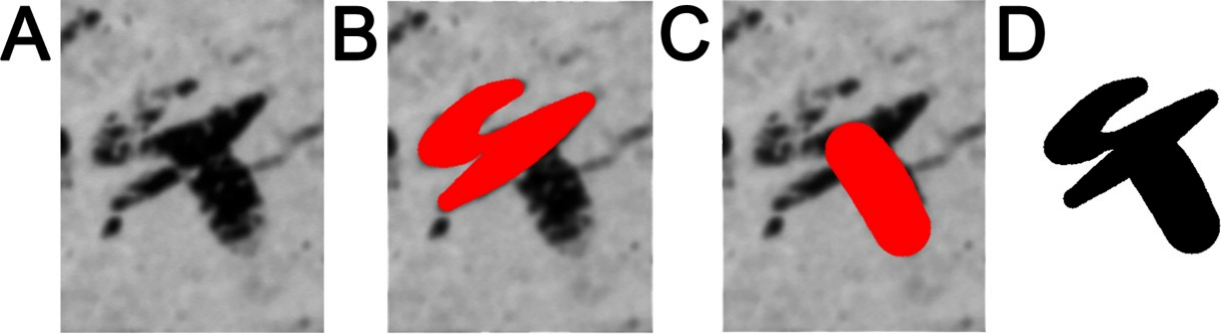
Restoration of a character *waw* in Arad Ostracon 24. (A) The original image. (B and C) reconstructed strokes. (D) The resulting character restoration. Images are courtesy of the Institute of Archaeology, Tel Aviv University and the Israel Antiquities Authority.

In the first step, a digital image of each inscription is segmented into characters, which are restored via a semi-automatic reconstruction procedure. The method is based on the representation of a given character as a union of individual strokes that are treated independently and later recombined. The stroke restoration imitates a reed/pen movement, optimizing the pen’s trajectory through manually sampled key-points. The restoration minimizes an energy functional, taking into account the adherence to the original image, the smoothness of the stroke, as well as certain properties of the reed radius. The minimization problem is solved by performing Gradient Descent iterations on a Cubic-Spline representation of the stroke. The end product of the reconstruction is a binary (black and white) image of each character, incorporating all its strokes; see [40] for additional details and Fig 4 for an example.

The second stage of the algorithm letter comparison relies on features extracted from the characters’ binary images, utilized in order to compare characters from different texts. The features in use are: SIFT [52], Zernike [53,54], DCT, kd-tree [55,56], image projections [57], L_1_ and CMI [58–60]. Additionally, for each feature, a respective distance is defined. Later on, all these distances are combined into a single, generalized feature vector. This vector describes each character by the degree of its proximity to all the characters, using all the features. Finally, a distance between any two characters is calculated according to the Euclidean distance between their generalized feature vectors.

The final, third stage of the algorithm addresses the question, “What is the probability that two given texts were written by the same writer?” The answer is achieved by posing an alternative null hypothesis H_0_ (“both texts were written by the same writer”) and attempting to reject it via an experiment. If the experiment’s outcome is unlikely (P≤0.1), we reject the H_0_ and conclude that the inscriptions were written by different individuals. Alternatively, if the H_0_ is probable (P>0.1), we remain agnostic. The experiment testing the H_0_ clusters a collection of characters of the same letter (e.g., *alep*) from two given inscriptions. Typically, if two different writers composed the documents, the clustering results would resemble the original inscriptions (i.e., most of the characters from the first inscription would be assigned to one cluster, while most of the characters from the second inscription would be assigned to another). Alternatively, if the documents were written by a single writer, we would expect the clustering results to be random. Moreover, if several different letters (e.g., *alep*, *he*, *waw*, etc.) are present, additional statistical significance would be gained by conducting independent experiments and combining the p-values via Fisher’s method [61]. The outcome represents the probability that H_0_ is true based on all the evidence at our disposal.

For additional details regarding the algorithm, see S1 File, Section 3.

### Algorithm #2: Writers’ identification via binary pixel patterns

This algorithm also aims at differentiating between writers in a given set of texts. The method is a major improvement of an algorithm previously published in [7]. The main alterations are a complete replacement of the p-values combination framework in order to account for dependencies between various features and letters, as well as a more aggressive filtering of the incoming input in order to prevent spurious results (for further details, see S1 File, Section 4).

The algorithm uses the same preliminary characters’ reconstruction procedure as Algorithm #1. Then, each individual binarized and segmented character is represented as a histogram of 512 overlapping black and white patches of 3×3 pixels, which will be denoted henceforth as *binary pixel patterns* [62,63]. In the process of comparing two given inscriptions under a “single writer” H_0_ hypothesis, we obtain a P via comparing the empirical distributions of occurrences of each patch for each letter under consideration. This is performed through Welch’s generalization [64] of a classic Student’s t-test [65]. The potentially hundreds of resulting P’s (for each binary pattern and each letter type) are combined using a dependency-correcting approach of Brown [66], including a computational improvement by Kost and McDermott [67]—producing a single P. The outcome represents the probability that a “single writer” hypothesis is true based on all the evidence at our disposal.

For additional details regarding the algorithm, see S1 File, Section 4.

## Results

**Fig 5:**
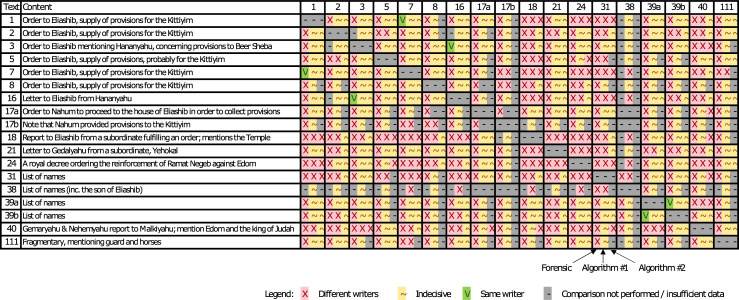
Comparison between different Arad ostraca via forensic and two algorithmic investigations. Three respective results are provided in each intersection in the table.

The independent outcomes of the three lines of investigation are presented in Fig 5; for more in-depth results, see S1 File, Sections 2–4. It should be stressed that by design, while the algorithmic methods are capable of distinguishing between *different* writers or otherwise remain indecisive, only the forensic expert is able to mark pairs of texts as written by the *same* writer.

The most important observation that can be construed from Fig 5 is that **according to the forensic analysis, the number of independent writers within the Arad corpus is at least 12 (!)**. Indeed, it can be easily seen from Fig 5 that Texts 5, 8, 17a, 21, 24, 31, 40, 111 were all created by different writers. Moreover, this property is maintained by adding either Text 1 or Text 7. Continuing this procedure, the same holds true when adding either 2, 3 or 16, as well as either 17b or 18, and either 39a or 39b. All in all, 24 sets of 12 inscriptions written by 12 different writers can be obtained in this fashion (e.g., 5, 8, 17a, 21, 24, 31, 40, 111, *1*, *3*, 18, 39a; OR 5, 8, 17a, 21, 24, 31, 40, 111, 7, 16, 18, 39b; etc.). The corresponding figures for the more “cautious” and thus less informative Algorithms #1 and #2 are a minimal number of 5 or 3 writers, not taking into account any information in the texts of the ostraca.

Another important remark is that **the forensic and the two algorithmic investigations exhibit no contradictions in their conclusions**. There are three cases where an *identicalness* of writers was established by the forensic expert; in all these cases the two algorithms remained agnostic.

Additional observations:

- The forensic handwriting analysis suggests a strong possibility that the two sides of Ostracon 39, listing names of individuals, were written by the same writer. On the other hand, Ostraca 31, 38 and 39 –all listing names and thus most probably composed at Arad–were all written by different writers (this evidence is also supported by Algorithm #1, separating 31 from 38). Thus, we obtain **at least three different writers at Arad**.
- The forensic analysis demonstrates a strong possibility that Ostraca 1 and 7 were composed by the same writer. This writer is one of the military officials requesting supplies for the *Kittiyim* mercenaries, possibly their direct Judahite commander (as will be assumed below), or liaison officer. On the other hand, it seems that among Ostraca 1, 2, 5, 7 and 8 (dealing with supplies to the *Kittiyim*), all texts except 1 and 7 were written by different hands (2 and 5 were also “separated” by Algorithm #1). Thus, **it is conceivable that leading the *Kittiyim* into desert reconnaissance missions was the responsibility of at least four literate Judahite military officers**.
- Finally, according to the forensic analysis, Ostraca 3 and 16 were probably composed by the same writer. Both of these inscriptions mention Hananyahu, possibly a quartermaster at Beer Sheba, ca. 25 km to the west of Arad, and were apparently written by him. Interestingly, both texts are two-sided, with the *verso* of Ostracon 3 containing only a few discernable characters [1], while the recently discovered *verso* of Ostracon 16 contains at least three lines of text [4,5].

## Discussion

The foremost methodological achievement of this paper is the thorough comparison of human vs. algorithmic analyses of ancient handwriting. The expertise of the forensic examiner produced significantly more “hands” separations compared to computational methods. Additionally, unlike the algorithmic techniques, human analysis allows for a detection of identicalness between writers. On the other hand, each result produced by the reported algorithms is accompanied by an easily interpretable statistical significance. Such a detail is inherently missing in the work of the forensic examiner, who relies on general assessments.

**Fig 6:**
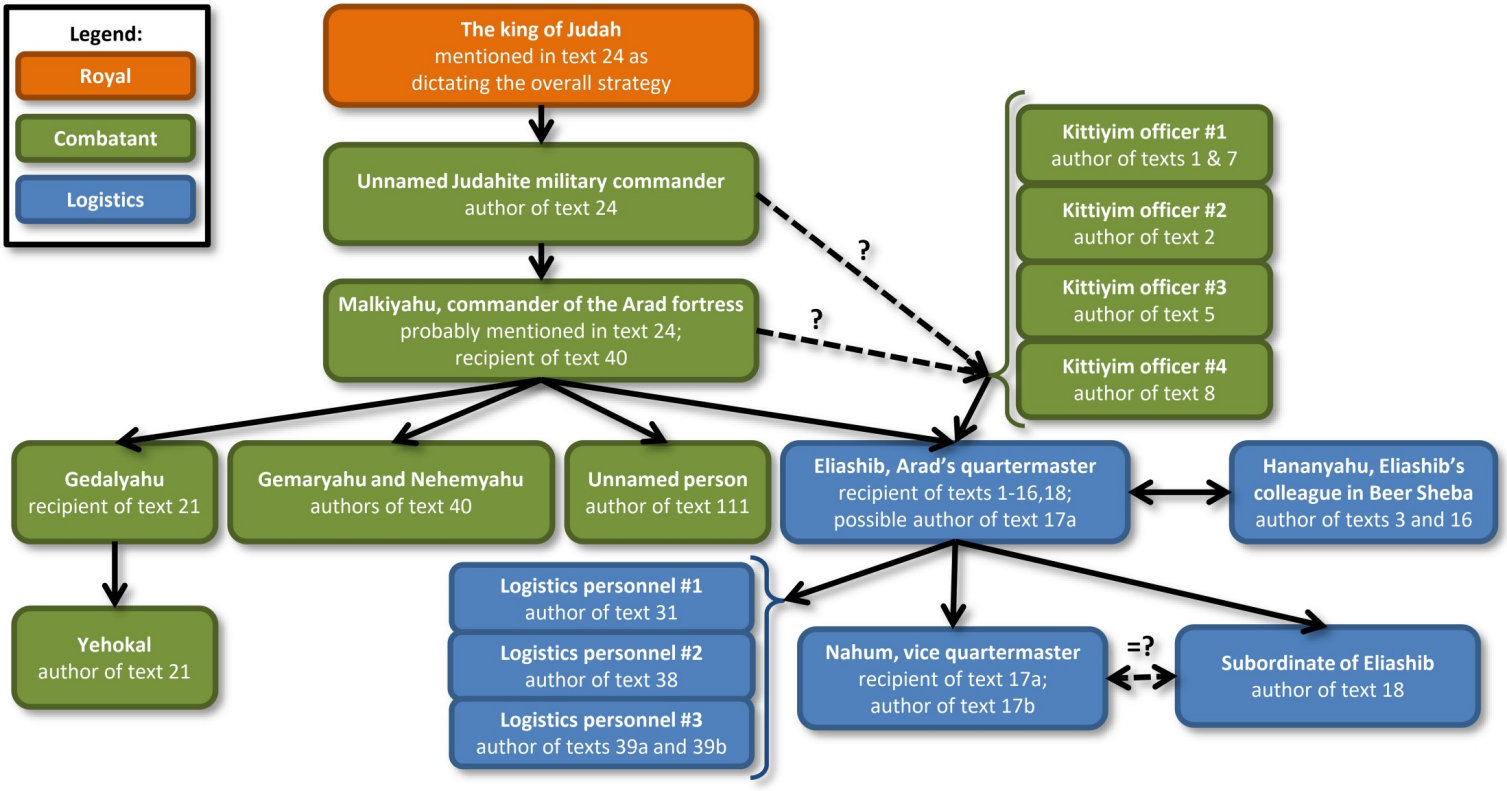
Possible reconstruction of relations between Arad inscriptions and different writers according to the forensic analysis. Differentiation between combatant and logistics officials is also indicated.

The notable result of **12 different “hands” out of 18 examined texts within the Arad corpus** (according to the forensic document examination), illuminates the issue of literacy in Judah at the end of the First Temple period. Even if some of the texts may have been sent to Arad from other locations in the region or farther away, there are still **at least 3 writers among the 20–30 military personnel stationed at this small fortress**. Additionally, a **minimum of 4 writers is observed among commanders of the Kittiyim unit/s**. A proposed reconstruction of the chain of command within the Judahite army (based on the information above, as well as on [1,6]; also see [2–5]), with written documents flowing from the king of Judah down to the vice quartermaster of the Arad fortress, is presented in Fig 6.

For broader significance, this textual evidence should be considered together with ostraca unearthed at other outposts in the southern periphery of Judah. We refer mainly to Horvat ‘Uza [68] (where 34 inscriptions were discovered, including a wisdom composition probably composed by a local scribe and reflecting a high degree of literacy [69]), Horvat Radum [68], Tel Malhata [70], Beer Sheba [71], Tel ‘Ira [72,73], Aroer [74], Tel Masos [75] and Kadesh Barnea [76]. The wealth of texts from the Negev (preserved because of dry weather conditions) can be supplemented by the military correspondence within the rich corpus from Lachish in the Shephelah [77] (the officer writing Lachish Ostracon 3 is seemingly offended by the suggestion that he is assisted by a scribe!), as well as by religious/cultic [12] and administrative [11,13,14] texts from other Judahite sites.

Widespread writing within the military, religious and civil bureaucracies hint at the **existence of an appropriate educational system in Judah at the end of the First Temple period** [10,78–82]. The unprecedented scribal activity during this era (cf. [83]) provides a **suitable literacy level and historical context for the composition and dissemination (including appreciation among the population) of several fundamental Judahite biblical texts**. We refer mainly to the Book of Deuteronomy and to the first version of the consolidated narrative presented in the Books of Joshua, Judges, Samuel and Kings–the so-called Deuteronomistic History [84,85]. These writings served as the law and "historical" platforms aimed at advancing the Judahite ideology and theology [85] at the end of 7^th^–beginning of the 6^th^ centuries BCE. Judging from archaeological data, the destruction of Jerusalem by Nebuchadnezzar in 586 BCE brought about decline if not cessation of this significant Hebrew literary activity in the southern highlands for the next four centuries [86].

## Supporting information

S1 File Supplementary material. (PDF)
