# Supplementary material for: Forensic document examination and algorithmic handwriting analysis of Judahite biblical period inscriptions reveal significant literacy level
Source: PLoS One. 2020 Sep 9;15(9):e0237962. doi: 10.1371/journal.pone.0237962 (PMC7480862; doi:10.1371/journal.pone.0237962)
Supplement: S1 File — (PDF) [file pone.0237962.s001.pdf]

Supplementary Material

Section 1: Datasets

Our experiments were conducted on two datasets, one used for the algorithmic and forensic analysis, and another for algorithmic verification. The first dataset pertained to Arad ancient Hebrew ostraca, dated to ca. 600 BCE, located on the southern frontier of the kingdom of Judah; see Fig 1. The inscriptions are described in detail in the main article and in [1,2,6]; examples can be seen in Fig 2. The examined texts were the sixteen ostraca 1, 2, 3, 5, 7, 8, 16, 17, 18, 21, 24, 31, 38, 39, 40 and 111 (see Table 1 for further details). Ostraca 17 and 39 contain writing on both sides of the potsherd, and were treated as separate texts 17a, 17b, 39a and 39b – totaling eighteen texts under investigation. The existence of *verso* in ostracon 16 [4,5] was still unknown to us upon conducting the analysis. We assumed that each of the inspected texts was written by a single writer. For forensic examination, the texts themselves, or their regular and multispectral images [4,5,35-37] were used.

Ancient as well as modern Hebrew is written from right to left, and the alphabet consists of 22 letters. The Latin transliteration of letter names used in this paper is: *alep, bet, gimel, dalet, he, waw, zayin, het, tet, yod, kap, lamed, mem, nun, samek, ayin, pe, sade, qop, resh, shin,* and *taw*. For algorithmic analysis, a semi-automatic restoration of the documents' characters [40] was performed. The seven letters we utilized were: *alep, he, waw, yod, lamed, shin* and *taw*, as they were the most prominent and simple to restore. In total, 427 characters were restored. For additional details and a complete dataset of the characters, see the main article, as well as [6]. The statistics for each relevant letter of the alphabet is presented at Table S1; the dataset can be downloaded at [41].

Table S1. Letter statistics for each Arad text under investigation.

	Alphabet letters						
**Text**	***Alep***	***He***	***Waw***	***Yod***	***Lamed***	***Shin***	***Taw***
**1**	4	5	3	7	3	3	8
**2**	6	3	3	5	3	1	7
**3**	2	4	5	4	4	3	3
**5**	5	3	1	3	4	2	4
**7**	1	2	1	4	6	8	5
**8**	2	1	2	1	4	4	2
**16**	6	3	9	5	10	3	2
**17a**	2	4	2	2	2	1	2
**17b**		1		2	1	1	2
**18**	2	4	4	5	6	6	3
**21**	5	4	6	6	12	5	2
**24**	9	10	5	8	4	4	7
**31**	3	7	6	4	1	1	
**38**	1	1	2	2	2	1	
**39a**	3	3	3	5	2	1	1
**39b**	3	1	1	4	1		
**40**	4	5	3	4		3	2
**111**	4	3	3	3	1	3	2

A second, modern Hebrew dataset, was used to estimate the False Positive and False Negative rates for the algorithmic methods we employed. It was not used for training or parameters' calibration purposes; in fact, the algorithms' designs aimed at minimizing the number of free parameters. The dataset contained handwriting samples collected from 18 writers, $i = 1,...,18$. Each individual filled in an alphabet table consisting of ten occurrences of each of the 22 letters in the alphabet (the number of letters in the alphabet and the letters themselves are the same in both ancient and modern Hebrew). These tables were scanned, and their characters were segmented. The modern Hebrew dataset can be downloaded at [42]; an example of a table produced by a single writer can be seen in Fig S1.

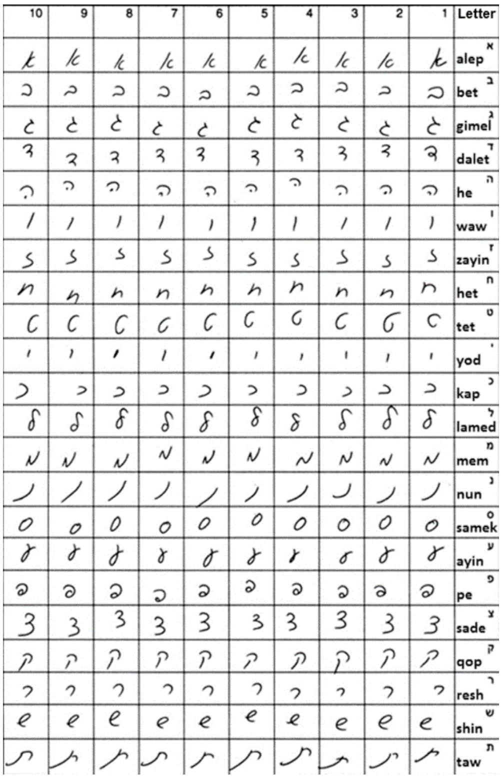

Fig S1: An example of a modern Hebrew alphabet table, produced by a single writer. 10 samples of each letter were provided.

From the obtained raw data, a series of "simulated" inscriptions were created. Due to the need to test both same-writer and different-writer scenarios, the data for each writer was split. Furthermore, in order to imitate a common situation in the ancient corpora, where the scarcity of data is prevalent, each simulated inscription used only 3 letters (i.e., 15 characters; 5 characters for each letter). In total, 252 inscriptions were "simulated" in the following manner:

- All the letters of the alphabet except for *yod* (as it is too small to be considered by some of the features) were split randomly into 7 groups (3 letters in each group) $g = 1,...,7$: *gimel, het, resh*; *bet*, *samek*, *shin*; *dalet*, *zayin*, *ayin*; *tet, lamed, mem*; *nun*, *sade*, *taw*; *he, pe, qop*; *alep, waw, kap*.
- For each writer i , and each letter belonging to group g , 5 characters were assigned into simulated inscription $S_{i,g,1}$, with the rest assigned to $S_{i,g,2}$.

In this fashion, for constant *i* and *g*, we can test if our algorithm arrives at wrong rejection of the null hypothesis ("same writer") for $S_{i,g,1}$ and $S_{i,g,2}$ (FP = "False Positive" error; 18 writers and 7 groups producing 126 tests in total). Additionally, for constant *g*, $1 \leq i \neq j \leq 18$, and $b, c \in \{1, 2\}$, we can test if our algorithms fail to correctly reject the null hypothesis for $S_{i,g,b}$ and $S_{j,g,c}$ (FN = "False Negative" error; $\frac{18x17}{2}x7x2x2 = 4284$ tests in total).

Section 2: Forensic Handwriting Analysis

The texts were collated and physically examined in various sites (see Table S2; cf. Table 1 for further details). When necessary, regular and/or multispectral images [4,5,35-37] of the same ostraca were utilized. The analysis was based on accepted forensic handwriting examination methods (see below).

Table S2: The physical location of the examined texts

Location	Texts
**The Israel Museum, Jerusalem**	1, 2, 3, 5, 7, 16, 17a, 17b, 18, 24, 39a, 39b, 40
**Israel Antiquities Authority, Beit Shemesh storage facility**	8, 38, 111
**Eretz Israel Museum, Tel Aviv**	21
**Examined via digital image**	31

Forensic Methods

The forensic handwriting examination process is divided into three steps: *analysis*, *comparison* and *evaluation*.

The analysis phase included a detailed examination of every single inscription according to the following features:

- **General appearance of the sherd**: size, form and type of pottery.
- **Writing style**: legibility, writing skill and flow and line quality.
- **Arrangement and use of space**: margins, spacing, alignment and formatting.
- **Size and proportions**: absolute and relative size of the writing and letters, alterations of size or height of upstrokes and downstrokes.
- **Slant**: general slant of the writing as well as an absolute and relative slant of letters.
- **Punctuation**: presence, form and position relative to the imaginary baseline of punctuation marks (or upper line in the case of Hebrew script).

- **Spacing**: spacing between letters, strokes, words and lines; relative position of letters vis-à-vis the preceding and following ones.
- **Alignment**: alignment of words and letters relative to an imaginary baseline.
- **Letter shapes and range of their variations within a script**: extraction of distinctive features.

The next phase was the comparison based upon the aforementioned features across different ostraca handwritings. In addition, consistent patterns, common for different inscriptions, were identified (i.e., the same combinations of letters, words, punctuation, etc.)

In what follows we present several characteristic features revealed by the examination.

General appearance of the ostraca

The examined ostraca have a rather large variance in their general appearance; see example in Fig 2 of the main article.

Writing style, arrangement and use of space, letter sizes

Fig S2 shows a comparison between Ostraca 21 and 31, which exemplifies several feature differences. For instance, our analysis indicated that Ostracon 21 (Fig S2 left) is probably written by a skillful writer. This observation is supported by the following points: the writing is organized, flowing and elegant; letters are small and aligned; there is a consistency in the compact spacing between letters and lines, the relative size of the letters, the relationship of the letters to the base line, the slant, etc. On the other hand, the writing in Ostracon 31 (Fig S2 right) is jumpy, its letters are squared with a mostly left-side inclination, and the spacing between letters and lines is not consistent.

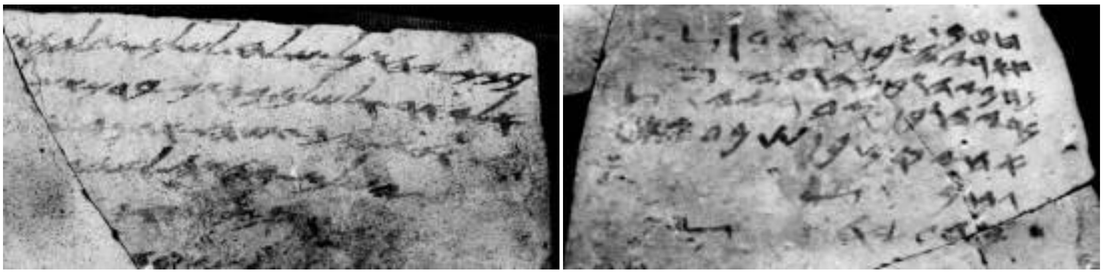

Fig S2. An example of significant differences in the general appearance, arrangement and space usage in two Arad ostraca. Left: Ostracon 21, right: Ostracon 31.

Slant

A demonstration of slant differences can be seen in Fig S3. Text 39b (Fig S3 left) is written with a slight right slant. In contrast, text 17a (Fig S3 right) is written with a strong right-side inclination, sometimes almost horizontal.

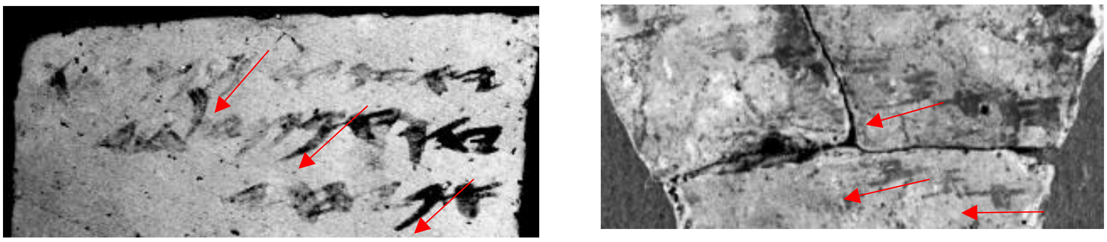

Fig S3. An example of a significant difference in the general slant of the script writing. Left: Text 39b, right: Text 17a; the arrows represent the slant direction.

Shape and location of punctuation marks

An example of punctuational differences can be observed in Fig S4. The dot (separating between words) has a round shape in Ostracon 1 (Fig S4 left), and a dash shape in Ostracon 24 (Fig S4 right).

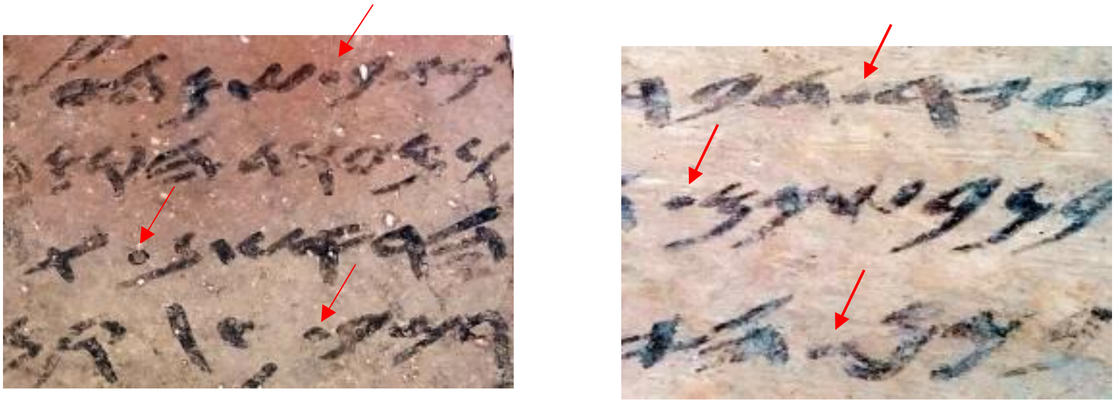

Fig S4. An example of significant difference in the punctuation, shape and location of punctuation marks. Left: Ostracon 1, right: Ostracon 24.

Letter shape

Some letters have distinct appearance in various Arad inscriptions. For instance, the entire corpus can be easily divided into two groups according to the shape of the letter *yod*, in the following manner:

- **Shape A** consists of three strokes: two horizontal and one vertical. The upper horizontal stroke starts on the left and ends at the upper edge of the vertical stroke, and the lower horizontal stroke crosses the vertical one. This form appears in Texts 1, 2, 3, 5, 7, 8, 16, 17a, 17b, 18, 21 and 24; see Fig S5, left.

- **Shape B** consists of four strokes: three horizontal and one vertical. The upper horizontal stroke is the same as in Shape A, the middle one is on the left side of the vertical one, and the lowest horizontal stroke is on the right side of the vertical one and starts at its bottom edge. This shape appears in Texts 31, 39a, 39b and 40; see Fig S5, right.

Notice that no ostracon has a mixed use of these two shapes.

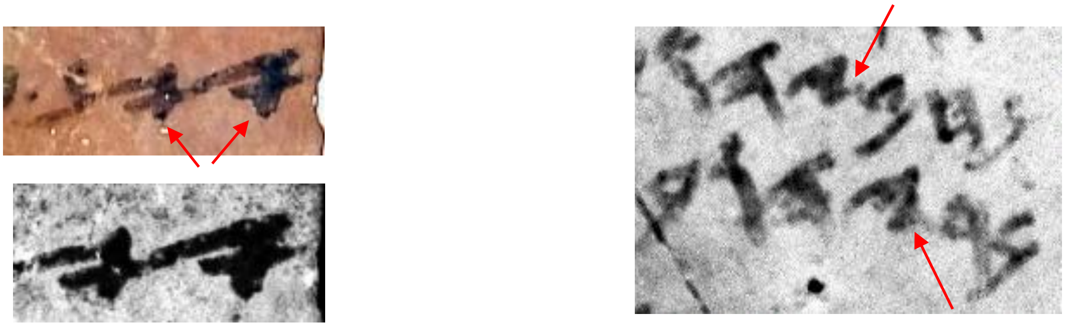

Fig S5. Significant differences between the two shapes of the letter *yod*. Left: Ostracon 1 - Shape A; right: Ostracon 31, Shape B.

In addition to the analysis of individual characters, sequences of several characters were also examined. For instance, in the case of *yod* that follows a *lamed*, the *yod* is written below the *lamed*'s baseline in Texts 1, 5, 7, 8, 17a, 18, 21, 24, 39a. However, in Texts 2, 3 and 16 the *yod* is located at the same baseline with the *lamed*. See examples in Fig S6.

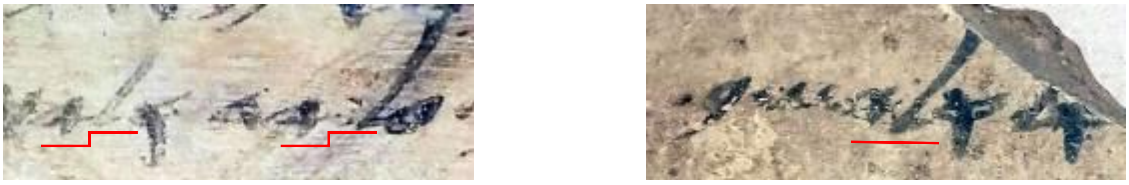

Fig S6. An example of significant difference in the relative position of the *yod* vis-à-vis the preceding *lamed*. Left: Ostracon 24, right: Ostracon 2.

Another example of an indicative sequence is a *yod* touching the following *he* in the middle of its vertical stroke (Fig S7 left). Contrastingly, the same sequence of *yod* and *he* could have been written with a consistent spacing, with the beginning of the upper stroke of the *yod* located approximately at the same level as the bottom edge of the vertical stroke in the following *he* (Fig S7 right; we acknowledge that this sequence, which is popular in names with the theophoric element *yhw* was often – but not always – compressed in such a way that the several graphemes touched one another).

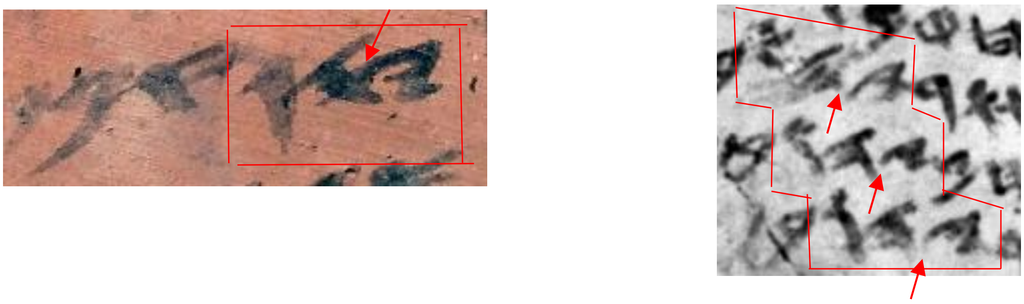

Fig S7. Example of consistent patterns in a relative spacing and position between sequential *yod* and *he*. Left: Ostracon 39, right: Ostracon 31.

Additional examples of shape differences in the letters *taw* and *nun* can be viewed respectively in Figs 3 and S8.

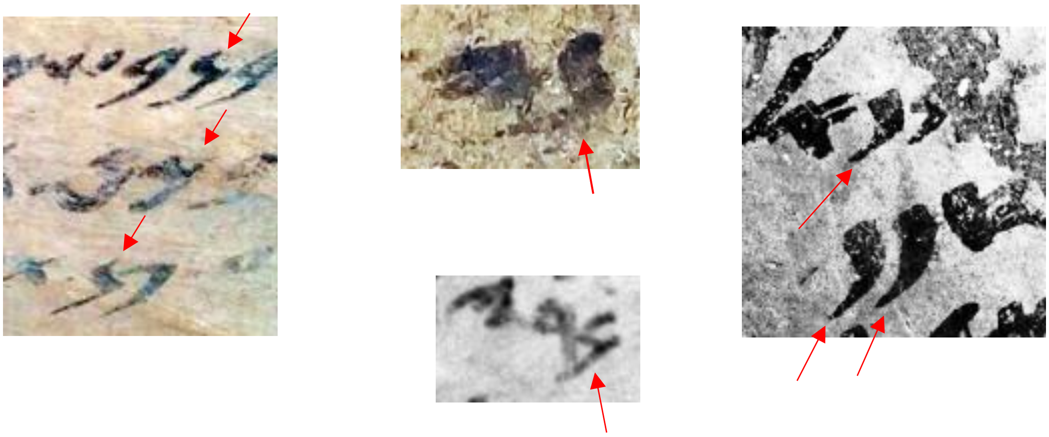

Fig S8. Examples of different shapes and slants in the letter *nun*. Left: Ostracon 24, top middle: Ostracon 7, bottom middle: Ostracon 31, right: Ostracon 38.

<u>Forensic classification</u>

Classification grades are based on the standard terminology guide for expressing conclusions of forensic document examiners [50,51]. For a detailed explanation regarding the possible grades, see Table S3.

Table S3. Scales of conclusions of forensic document examiners.

**Positive opinion**	
**Grade 1**	**Identification** (definite conclusion of identity). This is the highest degree of confidence that the two (or more) writings were written by the same person.
**Grade 2**	**Identification** (definite conclusion of identity) with only theoretical doubt due to certain limiting factors (in questioned or in known writing).
**Grade 3**	**Strong probability** that the two (or more) writings were written by the same person.
**Grade 4**	**Probable.** The evidence contained in the handwriting points rather strongly to the fact that the two (or more) writings were written by the same individual.
**Negative opinion**	
**Grade 5**	**Elimination.** This, like the definite conclusion of identity, is the highest degree of confidence expressed by the document examiner in handwriting comparisons that the two (or more) writings were not written by the same person.
**Grade 6**	**Strong probability did not.** This carries the same weight as strong probability on the identification side of the scale; that is that the writings were not written by the same person.
**Grade 7**	**Probably did not.** The evidence points rather strongly against the fact that the two (or more) writings were written by the same individual.
**Inconclusive opinion**	
**Grade 8**	**No conclusion.** It is used when there are significant limiting factors and the examiner does not lean one way or another.

Results

Using the methodology discussed above, the Arad texts were compared on a pair by pair basis. The results (grades) of the examination are summarized in Table S4. The texts' numbers head the rows and columns of the table, with the intersection cells providing the results.

Table S4. Results of forensic examination of the Arad texts. Grades 1 to 4 (in **green**) indicate the possibility of single writer's identity, with 1 the strongest and 4 the weakest degree of the positive identification. Grades 5 to 7 (in **red**) indicate different writer's identity, with 5 the strongest negative opinion and 7 the weakest one. Grade 8 (in **gray**) indicates an inability to draw a conclusion, in most cases due to insufficient data.

Text	1	2	3	5	7	8	16	17a	17b	18	21	24	31	38	39a	39b	40	111
**1**		6	6	6	3	6	7	6	7	6	6	6	5	8	5	5	5	7
**2**	6		8	7	6	6	8	7	6	6	6	7	5	8	5	5	5	7
**3**	6	8		7	6	6	4	7	7	6	6	7	5	8	5	5	5	7
**5**	6	7	7		6	7	7	7	7	6	7	7	5	8	5	5	5	7
**7**	3	6	6	6		7	7	6	6	7	7	6	5	8	5	5	5	7
**8**	6	6	6	7	7		7	6	7	7	7	7	5	8	5	5	5	7
**16**	7	8	4	7	7	7		7	7	7	7	7	5	8	5	5	5	7
**17a**	6	7	7	7	6	6	7		6	6	7	6	5	8	5	5	5	7
**17b**	7	6	7	7	6	7	7	6		8	7	7	5	8	5	5	5	7
**18**	6	6	6	6	7	7	7	6	8		6	6	5	8	5	5	5	7
**21**	6	6	6	7	7	7	7	7	7	6		7	5	8	5	5	5	7
**24**	6	7	7	7	6	7	7	6	7	6	7		5	8	5	5	5	7
**31**	5	5	5	5	5	5	5	5	6	5	5	5		6	5	5	5	6
**38**	8	8	8	8	8	8	8	8	8	8	8	8	6		7	7	6	8
**39a**	5	5	5	5	5	5	5	5	5	5	5	5	5	7		3	5	6
**39b**	5	5	5	5	5	5	5	5	5	5	5	5	5	7	3		5	6
**40**	5	5	5	5	5	5	5	5	5	5	5	5	5	6	5	5		6
**111**	7	7	7	7	7	7	7	7	7	7	7	7	6	8	6	6	6	

For further analysis, see the Results and Discussion sections of the main article.

Section 3: Writers' Identification via a Combination of Features

The goal of this algorithm is to distinguish between writers of different inscriptions, estimating the probability that two given inscriptions were written by the same writer. If the probability is lower than a pre-selected threshold, we consider the inscription to be written by two different writers, otherwise we remain agnostic.

A preliminary semi-automatic procedure, described in detail in [40], was performed in order to obtain a restoration of handwritten character strokes. The method is based on the representation of a given character as a union of individual strokes that are treated independently and later recombined. The stroke restoration imitates a reed-pen's movement, optimizing the pen's trajectory through manually sampled key-points. The restoration minimizes an energy functional, taking into account the adherence to the original image, the smoothness of the stroke, as well as certain properties of the reed radius. The minimization problem is solved by performing Gradient Descent iterations on a Cubic-Spline representation of the stroke. The end product of the reconstruction is a binary (black and white) image of each character, incorporating all its strokes; see Fig 4 for an example. These binarizations serve as an input to the algorithm.

The main steps of the algorithm are:

- **Feature extraction and distance calculation**: creation of feature vectors describing various aspects of the characters (e.g., angles between strokes and character profiles) and calculating the distance (similarity) between characters.
- **Testing the hypothesis that *two given inscriptions were written by the same writer***. Upon obtaining a suitable p-value (the significance level of the test, denoted as P), we reject the hypothesis of a single writer and accept the competing possibility, of two different writers; otherwise we remain undecided.

The previous version of the algorithm was presented in [6]. In the current paper, several changes were carried out. The main alterations, discussed in additional details below, are: lowering the *P* threshold to 0.l; k-medoid replacing the k-mean clustering algorithm; the non-homogeneity formula was changed in order to better represent the classes/inscriptions data; the calculations involving the count NC now take into account all the potential clustering outcomes, resulting in more accurate *P* calculations. Naturally, both the modern documents and the ancient Arad corpus results have now changed, and the results are provided below.

Description of the Algorithm

Feature extraction and distance calculation

Commonly, automatic comparison of characters relies upon features extracted from the characters' binary images. In this study, we adapted several well-established features from the domains of Computer Vision and Document Analysis. These features refer to aspects such as the character's overall shape, the angles between strokes, the character's center of gravity, as well as its horizontal and vertical projections. Some of these features correspond to characteristics commonly employed in traditional paleography [79].

The feature extraction process includes an initial step of the characters' standardization. This involves rotating the characters according to their line inclination, resizing them according to a pre-defined scale, and fitting the results into a padded (at least 10% on each side) square of size $a_L \times a_L$ (with $L = 1, ..., 22$ the index of the alphabet letter under consideration). On average, the resized characters were 300 by 300 pixels.

Subsequently, the proximity of two characters can be measured using each of the extracted features, representing various aspects of the characters. For each such feature, a *different* distance function is defined (later these distances are combined to create a vector representation of each character; see discussion below).

Table S5 provides a list of the features and distances which we employed, along with a description of their implementation details. Some of the adjustments (e.g., replacement of the L_2 norm with the L_1 norm) were required due to the large amount of noise present in our medium.

Table S5. Features and distances utilized by the writers' separation algorithm.

**Feature** [ref.]	**Implementation details**	**Distance implementation details**
**SIFT** [52]	For each character j, we use the normalized SIFT descriptors $\vec{d}_i \in \mathbb{R}^{128}$ (with $\left\Vert \vec{d}_i \right\Vert_2 = 1$) and the spatial locators $\vec{l}_i \in [1, a_L]^2$ for at most 40 significant key points $k_i = \left(\vec{d}_i, \vec{l}_i\right)$, according to the original SIFT implementation. The resulting feature is a set $f_j^{SIFT} = \left\{k_i\right\}_{i=1}^{40}$.	The distance between f_1^{SIFT} and f_2^{SIFT} is determined as follows:<br>• For each key point $k_i^1 \in f_1^{SIFT}$, find a matching key point $m_i^2 \in f_2^{SIFT}$ s. t. $m_i^2 = \arg\min_{\left(d_j^2, l_j^2\right) \in f_2^{SIFT}} dist(k_i^1, k_j^2)$; where $dist(k_i^1, k_j^2) = \arccos\left(\left\langle d_i^1, d_j^2 \right\rangle\right) \cdot \left\Vert l_i^1 - l_j^2 \right\Vert_2^2$. Thus, our definition enhances the original SIFT distance by adding spatial information.<br>• The one-sided distance is $D_{SIFT}^{1,2} = \underset{i}{median}\left\{dist(k_i^1, m_i^2)\right\}$.<br>• The final distance is $D_{SIFT}(1,2) = \left(D_{SIFT}^{1,2} + D_{SIFT}^{2,1}\right)/2$.
**Zernike** [53]	An off-the-shelf implementation was used [54]. Zernike moments up to the 5th order were calculated.	$D_{Zernike}$ is the *L1* distance between the Zernike feature vectors.
**DCT**	Standard MATLAB implementation was used.	D_{DCT} is the *L1* distance between the DCT feature vectors.
Kd-tree [55]	An off-the-shelf implementation was used [56]. Both orders of partitioning are employed (first height, then width and vice-versa)	$D_{Kd-tree}$ is the *L1* distance between the Kd-tree feature vectors.
**Image projections** [57]	The implementation results in cumulative distribution functions of the histogram on both axes.	D_{Proj} is the *L1* distance between the projections' feature vectors; this is similar to the Cramér–von Mises criterion (which uses *L2* distance).
**L1**	Existing character binarizations.	D_{L1} is the *L1* distance between the character images.
**CMI** [58-60]	Existing character binarizations, with values in $\{0,1\}$.	The CMI computes a difference between the averages of the foreground and the background pixels of $\mathcal{I}$, marked by a binary mask M, $CMI(M, \mathcal{I}) = \mu_1 - \mu_0$, where:<br>$\mu_k = mean\{\mathcal{I}(p,q) \mid M(p,q) = k\} \quad k = 0,1$<br>In our case, given character-binarizations B_1, B_2, the one-sided distance is $D_{CMI}^{1,2} = 1 - CMI(B_1, B_2)$.<br>The final distance is $D_{CMI}(1,2) = \left(D_{CMI}^{1,2} + D_{CMI}^{2,1}\right)/2$.

After the features are extracted, and the distances between the features measured, a combination of the various distances is required. In [6], a new combination technique was proposed. The main idea was to consider the distances of a given character from *all the other characters*, with respect to *all of the features* under consideration. That is, two characters closely resembling each other ought to have similar distances when compared to all other characters. Namely, they will both be at small distances from similar characters, and large distances from dissimilar characters. This observation leads to a notion of a *generalized feature vector*.

The generalized feature vector is defined by the following procedure (for each letter $L = 1,...,22$ in the alphabet). First, we define a *distance matrix* for each feature. For example, the SIFT distance matrix is:

$$U_{SIFT} = \begin{pmatrix} D_{SIFT}(1,1) & \cdots & D_{SIFT}(1,J_L) \\ \vdots & \ddots & \vdots \\ D_{SIFT}(J_L,1) & \cdots & D_{SIFT}(J_L,J_L) \end{pmatrix} = \begin{pmatrix} - & \vec{u}_{SIFT}^{1} & - \\ & \vdots & \\ - & \vec{u}_{SIFT}^{J_L} & - \end{pmatrix},$$

where J_L represents the total number of characters; $D_{SIFT}(i,j)$ is the SIFT distance between characters *i* and *j*; while $\vec{u}_{SIFT}^{i} = (D_{SIFT}(i,1)\cdots D_{SIFT}(i,J_L))$ is the vector of SIFT distances between the character *i* and all the others.

In addition, we denote the standard deviation of the elements of the matrix U_{SIFT} by $\sigma_{SIFT} = std\left\{D_{SIFT}(i,j) \mid (i,j) \in \{1,...,J_L\} \times \{1,...,J_L\}\right\}$. Matrices of all the other features ($U_{Zernike}$, U_{DCT}, and so forth) and their respective standard deviations ($\sigma_{Zernike}$, σ_{DCT}, etc.) are calculated in a similar fashion.

Eventually, each character k is represented by the following vector (of size $7 \cdot J_L$), concatenating the respective normalized row vectors of the distance matrices:

$$\vec{u}_k = \left(\frac{\vec{u}^k_{SIFT}}{\sigma_{SIFT}} \,\|\, \frac{\vec{u}^k_{Zernike}}{\sigma_{Zernike}} \,\|\, \frac{\vec{u}^k_{DCT}}{\sigma_{DCT}} \,\|\, \frac{\vec{u}^k_{Kd-tree}}{\sigma_{Kd-tree}} \,\|\, \frac{\vec{u}^k_{Proj}}{\sigma_{Proj}} \,\|\, \frac{\vec{u}^k_{L1}}{\sigma_{L1}} \,\|\, \frac{\vec{u}^k_{CMI}}{\sigma_{CMI}} \right) \in \mathbb{R}^{7 \cdot J_L}$$

In this fashion, each character is described by the degree of its kinship to all of the characters, using all the various features.

Finally, the distance between characters i and j is calculated according to the Euclidean distance between their generalized feature vectors:

$$chardist(i, j) = \left\| \vec{u}_i - \vec{u}_j \right\|_2 .$$

The main purpose of this distance is to serve as a basis for clustering at the next stage of the analysis.

Hypothesis Testing

At this stage we address the main question raised above: "*What is the probability that two given texts were written by the same writer*?" Commonly, similar questions are addressed by posing an *alternative* null hypothesis H_0 and attempting to reject it. In our case, for each pair of ostraca, the H_0 is: *both texts were written by the same writer*. This is performed by conducting an experiment (detailed below) and calculating the probability ($P \in [0,1]$) of affirmative answer to H_0. If this event is unlikely, i.e. $P \le 0.1$ (note the threshold is changed with respect to [6]), we conclude that the documents were written by two different individuals (i.e., reject H_0). On the other hand, if the occurrence of H_0 is probable ($P > 0.1$), we remain agnostic. We reiterate that in the latter case we cannot conclude that the two texts were written by a single writer.

The experiment, which is designed to test H_0, is comprised of several sub-steps (for additional details, see [6]):

- **Initialization**: We begin with two sets of characters of the same letter type (e.g., *alep*), denoted *A* and *B*, originating from two different texts.
- **Character clustering**: The union $A \cup B$ is a new, unlabeled set. This set is clustered into two classes, labeled I and II, using a brute-force (and not heuristic) implementation of k-medoids (k=2; note the clustering is not k-means as stated in [6]). The clustering utilizes the generalized feature vectors of the characters, and the distance *chardist*, defined above.
- **Non-homogeneity (NH) of the clustering**: The observed difference between the uniformity of the clustering results to the two original sets, *A* and *B*, is calculated as follows:

$$NH = NH_I = \left| \frac{\#(A \cap I)}{\# A} - \frac{\#(B \cap I)}{\# B} \right|,$$

  with # denoting a cardinality of a given set. It is easy to verify that the non-homogeneity score is well-defined, i.e., it is invariant to swap between I and II, $NH = NH_I = NH_{II}$. Note that this symmetry was enabled by a definition change with respect to [6].
- **Counting valid combinations**: We consider all the possible divisions of $A \cup B$ into two classes i and ii. The number of such valid combinations is denoted by NC. In fact, $NC = 2^{\#(A \cup B)} - 2$, since all the assignments of the characters to classes i and ii are considered, except for labeling all the characters as a single class. Note that this valid combinations' calculation is more inclusive than in [6].
- **Significance level calculation**: The p-value is calculated as:

$$P = \frac{\#\{i \mid NH_i \geq NH\}}{NC}.$$

I.e., *P* is the proportion of valid combinations with at least the same observed non-homogeneity. This is analogous to integrating over a tail of a probability density function.

The rationale behind this calculation is based on the scenario of *two writers* (negation of H_0). In such a case, we expect the k-medoids clustering to provide a sound separation of their characters, i.e., I and II would closely resemble A and B (or B and A). This would result in NH being close to 1. Furthermore, the proportion of valid combinations with $NH_i \geq NH$ will be meager, resulting in a low P. Therefore, the H_0 hypothesis would be justifiably rejected.

In the opposite scenario of a *single writer*:

- If a sufficient number of characters is present, there is an arbitrary low probability of receiving clustering results resembling A and B. In a common case, the NH will be low, which will result in high P.
- Alternatively, if the number of characters is low, the clustering may result in a high NH by chance. However, in this case NC would be low, and the P would remain high.

Either way, typically in this scenario we will not be able to reject the H_0 hypothesis.

Notes:

- We assume that each given text was written by a single writer. If multiple writers wrote the text, both H_0 and its negation should be altered. We do not cover such a case.
- The definition of P in sub-step 5 results in $P > 0$.
- Not every text provides a sufficient amount of characters for every type of letter in the alphabet. In our case, we do not perform comparisons for sets A and B such that: $(\#A=1)\&(\#B\leq 6)$ or $(\#B=1)\&(\#A\leq 6)$ or $(\#A=2)\&(\#B=2)$.

As specified, sub-steps 1-5 are applied to one specific letter of the alphabet (e.g., *alep*), present (in sufficient quantities) in the pair of texts under comparison. However, we can often gain additional statistical significance if several different letters (e.g., *alep*, *he*, *waw*, etc.) are represented in the

compared documents. In such circumstances, several independent experiments are conducted (one for each letter), resulting in corresponding P's. We combine the different values into a single P using the well-established Fisher method [61]. This end product represents the probability that H_0 is true based on all the evidence at our disposal.

Experimental details and results

As described above, our experiments were conducted on two datasets. A set of samples collected from contemporary writers of modern Hebrew allowed us to test the soundness of our algorithm. It was not used for parameter-tuning purposes, however, as the algorithm was kept as parameter-free as possible. The second dataset contained information from various Arad ancient Hebrew ostraca, dated to ca. 600 BCE.

Modern Hebrew script experiment

The Modern Hebrew experiment yielded **4.76% False Positive** and **2.66% False Negative** error rates. These results demonstrate the soundness of our algorithmic sequence. In fact, taking into account the 0.1 threshold, the empirical error rates may indicate "conservativeness" of our p-values estimation.

Arad ancient Hebrew script experiment

The results obtained by comparing the Arad texts are summarized in Table S6.

Table S6. Comparison between different Arad texts. A P≤0.1 highlighted in red indicates rejection of "single writer" hypothesis, hence accepting the "two different writers" alternative.

Text	1	2	3	5	7	8	16	17a	17b	18	21	24	31	38	39a	39b	40	111
1		0.23	0.75	0.86	0.29	0.25	0.45	0.29	0.64	0.01	0.71	0.01	0.03	0.60	0.41	0.21	0.02	0.45
2	0.23		0.28	0.09	0.11	0.85	0.27	0.14	0.90	0.09	0.02	0.12	0.01	0.38	0.70	0.01	0.06	0.86
3	0.75	0.28		0.85	0.11	0.68	0.81	0.47	0.99	0.03	0.55	0.80	0.51	0.12	0.99	0.91	0.07	0.69
5	0.86	0.09	0.85		0.87	0.31	0.46	0.47	0.21	0.06	0.63	0.14	0.03	0.13	0.17	0.42	0.27	0.46
7	0.29	0.11	0.11	0.87		0.22	0.81	0.16	3e-3	3e-3	0.36	4e-3	1e-3	0.01	0.13	0.73	0.03	0.01
8	0.25	0.85	0.68	0.31	0.22		0.65		0.03	0.06	0.41	2e-4	0.63	1.00	0.84	0.40	0.90	0.93
16	0.45	0.27	0.81	0.46	0.81	0.65		0.60	0.77	0.01	0.49	6e-4	1e-3	0.04	0.26	0.01	0.01	0.55
17a	0.29	0.14	0.47	0.47	0.16		0.60			0.36	0.98	0.23	0.54		1.00	0.81	0.42	1.00
17b	0.64	0.90	0.99	0.21	3e-3	0.03	0.77			0.15	0.12	0.18	0.24		0.85	0.69	0.15	0.92
18	0.01	0.09	0.03	0.06	3e-3	0.06	0.01	0.36	0.15		5e-5	7e-4	0.02	0.03	0.60	0.85	0.21	0.57
21	0.71	0.02	0.55	0.63	0.36	0.41	0.49	0.98	0.12	5e-5		0.04	5e-4	0.77	0.10	2e-4	0.02	0.12
24	0.01	0.12	0.80	0.14	4e-3	2e-4	6e-4	0.23	0.18	7e-4	0.04		2e-6	2e-3	0.53	0.25	7e-6	0.43
31	0.03	0.01	0.51	0.03	1e-3	0.63	1e-3	0.54	0.24	0.02	2e-3	2e-6		0.07	0.32	0.94	0.39	0.68
38	0.60	0.38	0.12	0.13	0.01	1.00	0.04			0.03	0.77	5e-4	0.07		0.37	0.81	0.46	0.58
39a	0.41	0.70	0.99	0.17	0.13	0.84	0.26	1.00	0.85	0.60	0.10	0.53	0.32	0.37		0.93	0.01	0.73
39b	0.21	0.01	0.91	0.42	0.73	0.40	0.01	0.81	0.69	0.85	2e-4	0.25	0.94	0.81	0.93		0.58	0.28
40	0.02	0.06	0.07	0.27	0.03	0.90	0.01	0.42	0.15	0.21	0.02	7e-6	0.39	0.46	0.01	0.58		0.19
111	0.45	0.86	0.69	0.46	0.01	0.93	0.55	1.00	0.92	0.57	0.12	0.43	0.68	0.58	0.73	0.28	0.19	

As can be seen from Table S6, 44 separations out of 149 comparisons were achieved, with the p-values as low as ~2×10^{-6}. Additionally, we can observe two pair-wise distinct "quintuplets" of texts: I) **7, 18, 24, 31** and **38**; II) **16, 18, 24, 31** and **38**. In other words, if the five pair-wise distinct writer identities are indeed true, then at least five different hands produced the corpus of Arad inscriptions. The existence of two such combinations indicates the high probability that the corpus indeed contains at least five different writers (the probability of obtaining at least one pair-wise distinct "quintuplet" of texts on a random graph with a configuration similar to Arad, with edge probability of 0.1, is 1×10^{-7}).

It will be stressed that the separated Inscriptions 31 and 38 contain lists of names, and their writers were most likely located at the Arad fort itself. This implies the composition by writers who were not professional scribes. For further analysis, see the Results and Discussion sections of the main article.

Section 4: Writers' Identification via Binary Pixel Patterns

This writer identification analysis is performed independently, not only on the level of a single letter as in the previous section, but also on the level of individual feature. In addition, the algorithm uses an entirely different set of features, which are the 512 binary (black & white) pixel patterns [62,63] of size 3×3 (e.g., a patch 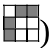). The basic algorithmic flow is:

- **Feature Extraction and Histograms Calculation**: creating histograms of frequencies of different features in any given character (e.g., 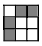 may constitute 8.3% of the patches).
- **Testing the hypothesis that *two given inscriptions were written by the same writer***. Extract p-value for identicalness for each letter type and each feature via Welch's generalization [64] of a classic Student's t-test [65]. The potentially hundreds of resulting P (for each binary pattern and each letter type) are combined using a dependency-correcting approach of Brown [66], including a computational improvement by Kost and McDermott [67] - producing a single P.

The resulting "meta" p-value represents the probability that a "single writer" hypothesis is true based on all the evidence at our disposal.

The overall method is a major improvement of an algorithm previously published in [7]. The main alterations are a complete replacement of the p-values combination framework (2nd stage of the algorithm) in order to account for possible statistical inter-dependencies between various features and letters. A more aggressive filtering of the incoming input was added in order to prevent spurious results.

Description of the Algorithm

Preliminary Remarks

Similarly to the previous section, we use the common statistical convention of defining a "null hypothesis" H_0 and trying to *disprove* it. Again, H_0 is "two given inscriptions were written by the same writer." The probability for this event is the p-value, which will be estimated via the algorithm. If the p-value is *lower* than a pre-defined threshold, H_0 is rejected, and the competing hypothesis of "two different writers" is declared valid. On the other hand, an inability to reject the null hypothesis does not indicate its validity. In such a case we remain agnostic, not being able to say anything regarding the documents' writers.

Prior Assumptions

We begin with two images of different inscriptions, denoted as I and J. The algorithm operates based on information derived at a character level. We assume that the inscriptions' characters are binarized and segmented into images $I_{i_l}^l$ ($i_l = 1,...,M_l$, representing the instances of the letter l within I); and $J_{j_l}^l$ ($j_l = 1,...,N_l$, representing the instances of the same letter l within J), belonging to appropriate letters ($l = 1,...,L$). In the current research, the binarization and segmentation was performed automatically for modern Hebrew, and in semi-manual fashion for ancient Hebrew documents; see above and in [40] for further details. The resulting characters' images are normalized to obtain an area of 17,000 pixels (see [7] for details), and padded with a 1-pixel white border on each side.

Histogram creation for each character

Our features are the 3×3 binary pixel patterns, i.e., image patches of the individual characters. For additional information on pixel patterns, see the examples in [62,63]. There are 2^9=512 optional patches of the requested size. All such possible patches are extracted from the images $I_{i_l}^l$ and $J_{j_l}^l$, in

order to create normalized patch histograms (counting frequencies of patch occurrences), $H^{l}_{i_l}(p)$ and $G^{l}_{j_l}(p)$, respectively (with $p = 1,\ldots,512$).

A simple, yet illustrative, example of two such images and their respective histograms is seen in Table S7. Remarkably, despite a similar overall shape of the character and a difference of only two pixels in the character images, 16 out of 19 meaningful histogram entities are different.

Table S7. Illustrative example of features (patches) and their histograms for two different characters. Only the meaningful (non-zero for at least one character) histogram entries are provided; discrepancies between the two histograms are marked in red.

Patches	Character images, patch counts and frequency histograms			
	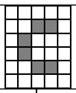		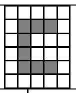	
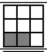	1	0.083	1	0.083
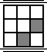	1	0.083	0	0
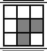	0	0	1	0.083
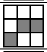	1	0.083	0	0
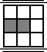	2	0.167	2	0.167
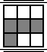	0	0	1	0.083
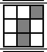	1	0.083	0	0
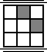	1	0.083	0	0
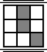	1	0.083	0	0
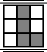	0	0	1	0.083
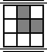	0	0	1	0.083
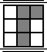	0	0	1	0.083
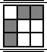	1	0.083	0	0
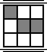	1	0.083	0	0
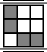	1	0.083	0	0
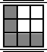	0	0	1	0.083
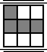	0	0	1	0.083
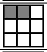	1	0.083	1	0.083
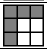	0	0	1	0.083

We stress that the histograms only serve for normalization purposes. In the following, the histograms themselves will **not** be compared. Instead, the comparison will take place on an individual feature (patch) level, across different characters.

Same Writer Statistics Derivation

The experiments are performed in the following fashion: for given inscriptions' images I and J with $I \neq J$:

- Empty $PVALS$ and ROS arrays, to be utilized are initialized.
- For each letter $l = 1,...,L$, with at least 4 character instances present in each inscription (i.e., $M_l \geq 4$ and $N_l \geq 4$; a more aggressive filtering of the input compared to [7]):
    - For each patch $p = 1,...,512$, with at least 4 nonzero terms present in each histogram:
        - The p-values $PVALS$ are calculated via Welch's generalization [64] of Student's t-test [65] between the two samples $S_p^l = \left\{H_{i_l}^l(p)\right\}_{i_l=1}^{M_l}$ and $T_p^l = \left\{G_{j_l}^l(p)\right\}_{j_l=1}^{N_l}$, setting $pval_p^l = Welch\left(S_p^l, T_p^l\right)$ and appending $pval_p^l$ to $PVALS$.
        - The corresponding correlations ROS are computed for *specific feature* p, by taking into account mean histogram values $U_p = \left(mean\left\{H_{i_l}^l(p)\right\}_{i_l=1...M_l}\right)_{l \in L}$ and $V_p = \left(mean\left\{G_{j_l}^l(p)\right\}_{j_l=1...N_l}\right)_{l \in L}$ for *all* letters L common to I and J, setting $\rho_p^l = \rho_P = cor(U_p, V_p)$ and appending ρ_p^l to ROS.

    (Note this stage is a complete replacement of the corresponding stage in [7], which uses Kolmogorov-Smirnov test and does not take into account possible correlations.)
- If the $PVALS$ array is empty (i.e., no experiments were performed due to the scarcity of data), or if $I = J$, set: $SameWriterP(I, J) = SameWriterP(J, I) = 1$.

- Otherwise utilize the Brown's method [66], including Kost's and McDermott's correction [67] for combination of possibly correlated p-values on $PVALS$, and utilizing the correlations ROS, setting: $SameWriterP(I,J) = SameWriterP(J,I) = Brown(PVALS, ROS)$.

  (Note this stage is a complete replacement of the corresponding stage in [7], which assumed un-correlatedness and made do with a basic Fisher combination [61] of $PVALS$.)

$SameWriterP(I,J)$ represents the deduced probability of having the *same* writer in both I and J (the H_0 hypothesis). As in the previous section, a threshold of $pval \le 0.1$ was used for rejecting the H_0.

Experimental details and results

Modern Hebrew script experiment

The modern Hebrew experiment yielded **0% (!) False Positive** and **5.18% False Negative** error rates. These results demonstrate the soundness of our algorithmic sequence. As in the previous section, taking into account the 0.1 threshold, the empirical error rates may indicate "conservativeness" of our p-value estimation.

Arad ancient Hebrew script experiment

The results obtained by comparing the Arad texts are summarized in Table S8. For further analysis, see the Results and Discussion sections of the main article.

Table S8. Comparison between different Arad texts. A P≤0.1 highlighted in red, indicates rejection of "single writer" hypothesis, hence accepting the "two different writers" alternative.

Text	1	2	3	5	7	8	16	17a	17b	18	21	24	31	38	39a	39b	40	111
1		0.15	0.15	0.27	0.18		0.24	0.25		0.08	0.23	0.04	0.02		0.14	0.14	0.14	0.38
2	0.15		0.49	0.31	0.18		0.16			0.17	0.14	0.21	0.20		0.34	0.17	0.14	0.46
3	0.15	0.49		0.38	0.39	0.38	0.34	0.85		0.13	0.40	0.33	0.14		0.59	0.37	0.08	
5	0.27	0.31	0.38		0.42	0.44	0.14			0.004	0.11	0.05					0.50	0.27
7	0.18	0.18	0.39	0.42		0.29	0.22			0.03	0.19	0.10	0.08		0.21	0.39	0.24	
8			0.38	0.44	0.29		0.14			0.12	0.17	0.32						
16	0.24	0.16	0.34	0.14	0.22	0.14				0.02	0.33	0.15	0.02		0.25	0.50	0.22	0.28
17a	0.25		0.85							0.69	0.43	0.33	0.47				0.08	
17b																		
18	0.08	0.17	0.13	0.004	0.03	0.12	0.02	0.69			0.05	0.05	0.26		0.15	0.14	0.05	
21	0.23	0.14	0.40	0.11	0.19	0.17	0.33	0.43		0.05		0.09	0.14		0.45	0.42	0.17	0.23
24	0.04	0.21	0.33	0.05	0.10	0.32	0.15	0.33		0.05	0.09		0.05		0.31	0.43	0.04	0.47
31	0.02	0.20	0.14		0.08		0.02	0.47		0.26	0.14	0.05			0.30	0.13	0.04	
38																		
39a	0.14	0.34	0.59		0.21		0.25			0.15	0.45	0.31	0.30			0.31	0.05	
39b	0.14	0.17	0.37		0.39		0.50			0.14	0.42	0.43	0.13		0.31		0.12	
40	0.14	0.14	0.08	0.50	0.24		0.22	0.08		0.05	0.17	0.04	0.04		0.05	0.12		0.47
111	0.38	0.46		0.27			0.28				0.23	0.47					0.47	

For further analysis, see the results and discussion sections of the main article.

References

1. Aharoni Y. Arad Inscriptions. Jerusalem: Israel Exploration Society; 1981 [1975, Hebrew].

2. Na'aman N. Textual and historical notes on the Eliashib archive from Arad. Tel Aviv. 2011; 38.1: 83–93.

3. Lemaire A. Inscriptions hébraïques, tome I: Les ostraca. Paris: Les éditions du Cerf. 1977. pp 230–231.

4. Faigenbaum-Golovin S, Mendel-Geberovich A, Shaus A, Sober B, Cordonsky M, Levin D, et al. Multispectral imaging reveals biblical-period inscription unnoticed for half a century. PLOS ONE. 2017; 12.6: e0178400. https://doi.org/10.1371/journal.pone.0178400

5. Mendel-Geberovich A, Shaus A, Faigenbaum-Golovin S, Sober B, Cordonsky M, Piasetzky E, et al. A brand new old inscription: Arad ostracon 16 rediscovered via multispectral imaging. Bull Am Schools Orient Res. 2017; 378: 113-125. https://doi.org/10.5615/bullamerschoorie.378.0113

6. Faigenbaum-Golovin S, Shaus A, Sober B, Levin D, Na'aman N, Sass B, et al. Algorithmic handwriting analysis of Judah's military correspondence sheds light on composition of biblical texts. Proc Natl Acad Sci U S A. 2016; 113.17: 4664-4669. https://doi.org/10.1073/pnas.1522200113

7. Shaus A, Turkel E. Writer identification in modern and historical documents via binary pixel patterns, Kolmogorov-Smirnov test and Fisher's method. J Imaging Sci Technol. 2017; 61.1: 010404-1–010404-9. https://doi.org/10.2352/J.ImagingSci.Technol.2017.61.1.010404

8. Faigenbaum-Golovin S, Levin D, Piasetzky E, Finkelstein I. Writer characterization and identification in short modern and historical documents: Reconsidering paleographic tables. Proceedings of the 19th ACM Symposium on Document Engineering (DocEng2019), 23:1–23:4.

9. Schmid K. The Old Testament: A Literary History. Minneapolis: Fortress; 2012.

10. Rollston CA. Writing and Literacy in the World of Ancient Israel: Epigraphic Evidence from the Iron Age. Atlanta: Society of Biblical Literature; 2010.

11. Naveh JA. Hebrew letter from the seventh century B.C. Israel Exploration Journal. 1960; 10.3: 129-139.

12. Barkay G, Vaughn AG, Lundberg MJ, Zuckerman B. The amulets from Ketef Hinnom: A new edition and evaluation. Bull Am Schools Orient Res. 2004; 334: 41–71.

13. Faigenbaum-Golovin S, Rollston CA, Piasetzky E, Sober B, Finkelstein I. The Ophel (Jerusalem) ostracon in light of new multispectral images. Semitica. 2015; 57: 113-137. https://doi.org/10.2143/SE.57.0.3115458

14. Mendel-Geberovich A, Faigenbaum-Golovin S, Shaus A, Sober B, Cordonsky M, Piasetzky E, et al. A renewed reading of Hebrew ostraca from cave A-2 at Ramat Beit Shemesh (Nahal Yarmut), based on multispectral imaging. Vetus Testamentum. 2019; 69: 682–701. https://doi.org/10.1163/15685330-00001370

15. Pessanha S, Manso M, Guilherme A, Costa M, Carvalho ML. Investigation of historical documents for forensic purposes by x-ray fluorescence spectrometry. Surf. Interface Anal. 2010; 42: 419–422.

16. Srihari SN, Cha S-H, Arora H, Lee S, Individuality of handwriting, J. Forensic Sci. 2002, 47: 1-17.

17. Srihari SN, Shi Z. Forensic handwritten document retrieval system. Proceedings of the First International Workshop on Document Image Analysis for Libraries. 2004: 1-7.

18. Srihari S. Handwriting Recognition, Automatic. In: Brown K, editor. Encyclopedia of Language and Linguistics, 2nd edition, vol. 5; 2006. pp. 203–211.

19. Srihari S, Huang C, Srinivasan H, On the discriminability of the handwriting of twins, J. Forensic Sci., 2008, 53.2: 430-446.

20. Birajdar GK, Mankar VH. Digital image forgery detection using passive techniques: A survey. Digital Investigation. 2013; 10.3: 226-245.

21. Warif NBA, Wahab AWA, Idris MYI, Ramli R, Salleh R, Shamshirband S, et al. Copy-move forgery detection: survey, challenges and future directions. Journal of Network and Computer Applications. 2016; 75: 259-278.

22. Panagopoulos M, Papaodysseus C, Rousopoulos P, Dafi D, Tracy S. Automatic writer identification of ancient Greek inscriptions. IEEE Trans Pattern Anal Mach Intell. 2009; 31.8: 1404-1414.

23. Papaodysseus C, Rousopoulos P, Giannopoulos F, Zannos S, Arabadjis D, Panagopoulos M, et al. Identifying the writer of ancient inscriptions and byzantine codices. A novel approach. Comput Vis Image Underst. 2014; 121: 57-73.

24. De Stefano C, Maniaci M, Fontanella F, di Freca AS. Reliable writer identification in medieval manuscripts through page layout features: The “Avila” Bible case. Eng Appl Artif Intell. 2018; 72: 99-110.

25. Ball GR, Srihari SN, Stritmatter R. Writer verification of historical documents among cohort writers. Proceedings of the 12th International Conference on Frontiers in Handwriting Recognition Frontiers in Handwriting Recognition (ICFHR 2010); 314-319.

26. Fecker D, Asi A, Pantke W, Märgner V, El-Sana J, Fingscheidt T. Document writer analysis with rejection for historic Arabic manuscripts. Proceedings of the 14th International Conference on Frontiers in Handwriting Recognition (ICFHR 2014); 743-748.

27. Fecker D, Asi A, Märgner V, El-Sana J, Fingscheidt T. Writer identification for historical Arabic documents. Proceedings of the 22nd International Conference on Pattern Recognition (ICPR 2014); 3050-3055.

28. Asi A, Abdalhaleem A, Fecker D, Märgner V, El-Sana J. On writer identification for Arabic historical manuscripts. Int J Doc Anal Recognit. 2017; 20.3: 173-187.

29. Dhali MA, He S, Popović M, Tigchelaar E, Schomaker L. A digital palaeographic approach towards writer identification in the Dead Sea scrolls. Proceedings of the 6th International Conference on Pattern Recognition Applications and Methods (ICPRAM 2017); pp. 693-702.

30. Bar-Yosef I, Beckman I, Kedem K, Dinstein I. Binarization, character extraction, and writer identification of historical Hebrew calligraphy documents. Int J Doc Anal Recognit. 2017; 9.2: 89-99.

31. Wolf L, Littman R, Mayer N, German T, Dershowitz N, Shweka R, et al. Identifying Join Candidates in the Cairo Genizah. Int J Comput Vis. 2011; 94.1: 118–135.

32. Davis T, The practice of handwriting identification. The Library: The Transactions of the Bibliographical Society 2007, 8.3: 251-276.

33. Schomaker LRB. Writer identification and verification. In: N. Ratha, V. Govindaraju, editors. Advances in Biometrics: Sensors, Systems and Algorithms. London: Springer-Verlag; 2007. pp. 247-264.

34. Sreeraj M, Idicula SM. A survey on writer identification schemes. Int J Comput Appl. 2011; 26.2: 23-33.

35. Faigenbaum S, Sober B, Shaus A, Moinester M, Piasetzky E, Bearman G, et al. Multispectral images of ostraca: Acquisition and analysis. J Archaeol Sci. 2012; 39.12: 3581–3590. https://doi.org/10.1016/j.jas.2012.06.013

36. Sober B, Faigenbaum S, Beit-Arieh I, Finkelstein I, Moinester M, Piasetzky E, et al. Multispectral imaging as a tool for enhancing the reading of ostraca. Palest Explor Q. 2014; 146.3: 185-197. https://doi.org/10.1179/0031032814Z.000000000101

37. Faigenbaum S, Sober B, Finkelstein I, Moinester M, Piasetzky E, Shaus A, et al. Multispectral imaging of two Hieratic inscriptions from Qubur el-Walaydah. Egypt and the Levant 2014; 24: 349-353. https://doi.org/10.1553/s349

38. Nir-El Y, Goren Y, Piasetzky E, Moinester M, Sober B. X-ray fluorescence (XRF) measurements of red ink on a Tel Malhata ostracon. In: Beit-Arieh I, Freud L, editors. Tel Malhata: A Central City in the Biblical Negev (Tel Aviv University Monograph Series 32). Tel Aviv University; 2015; pp. 510-513.

39. Shaus A, Sober B, Tzang O, Ioffe Z, Cheshnovsky O, Finkelstein I, et al. Raman binary mapping of Iron Age ostracon in unknown material composition and high fluorescence setting – A proof of concept. Archaeometry. 2019; 61.2: 459-469. https://doi.org/10.1111/arcm.12419

40. Sober B, Levin D. Computer aided restoration of handwritten character strokes. Comput Aided Des. 2017; 89: 12-24.

41. Arad Ancient Hebrew dataset, figshare (CC BY 4.0 license): https://doi.org/10.6084/m9.figshare.11423181.v1

42. Modern Hebrew dataset, figshare (CC BY 4.0 license): https://doi.org/10.6084/m9.figshare.11423352.v1

43. Morris RN. Forensic Handwriting Identification: Fundamental Concepts and Principles. London and San Diego: Academic Press; 2000.

44. Osborn AS. Questioned Documents. Albany: Boyd Printing Company; 1929.

45. Conway JVP. Evidential Documents. Springfield: Thomas; 1959.

46. Harrison WR. Suspect Documents: Their Scientific Examination. New York: Praeger; 1958.

47. Hilton O. Scientific Examination of Questioned Documents, New York: Elsevier; 1982.

48. Huber RA, Headrick AM. Handwriting Identification Facts and Fundamentals. Boca Raton: CRC Press; 1999.

49. ASTM E2290-07a, Standard Guide for Examination of Handwritten Items (Withdrawn 2016). West Conshohocken, PA: ASTM International; 2007. Available from: www.astm.org.

50. McAlexander TV, Beck J, Dick R. The Standardization of Handwriting Opinion Terminology (ASTM Standard E1658). J Forensic Sci. 1991; 36.2: 311-319.

51. ASTM E1658-08, Standard Terminology for Expressing Conclusions of Forensic Document Examiners (Withdrawn 2017), West Conshohocken, PA: ASTM International; 2008. Available from: www.astm.org

52. Lowe DG. Distinctive image features from scale-invariant keypoints. Int J Comput Vis. 2004; 60: 91–110.

53. Tahmasbi A, Saki F, Shokouhi SB. Classification of benign and malignant masses based on Zernike moments. Comput Biol Med. 2011; 41: 726–735.

54. Tahmasbi A. Zernike moments. 2012. Available from: www.mathworks.com/matlabcentral/fileexchange/38900-zernike-moments

55. Sexton A, Todman A, Woodward K. Font recognition using shape-based quadtree and kd-tree decomposition. Proceedings of the 3rd International Conference on Computer Vision, Pattern Recognition and Image Processing (CVPRIP 2000); 212–215.

56. Armon S. Descriptor for shapes and letters (feature extraction). 2012. Available from: www.mathworks.com/matlabcentral/fileexchange/35038-descriptor-for-shapes-andletters-feature-extraction

57. Trier ØD, Jain AK, Taxt T. Feature extraction methods for character recognition—A survey. Pattern Recognit. 1996; 29: 641–662.

58. Shaus A, Turkel E, Piasetzky E. Quality evaluation of facsimiles of Hebrew First Temple period inscriptions. Proceedings of the 10th IAPR International Workshop on Document Analysis Systems (DAS 2012); 170–174. https://doi.org/10.1109/DAS.2012.70

59. Shaus A, Turkel E, Piasetzky E. Binarization of First Temple period inscriptions - Performance of existing algorithms and a new registration based scheme. Proceedings of the 13th International Conference on Frontiers in Handwriting Recognition (ICFHR 2012); 641–646. https://doi.org/10.1109/ICFHR.2012.187

60. Shaus A, Faigenbaum-Golovin S, Sober B, Turkel E, Piasetzky E. Potential contrast - A new image quality measure. Proceedings of the IS&T International Symposium on Electronic Imaging 2017, Image Quality and System Performance XIV Conference (IQSP 2017); 52-58. https://doi.org/10.2352/ISSN.2470-1173.2017.12.IQSP-226

61. Fisher RA. Statistical Methods for Research Workers. Edinburgh: Oliver and Boyd; 1925.

62. Akiyama T, Miyamoto N, Oguro M, Ogura K. Faxed document image restoration method based on local pixel patterns. Proceedings of Photonics West '98 Electronic Imaging Symposium. 1998; 253-262.

63. Ratnakar V, RAPP, lossless image compression with runs of adaptive pixel patterns. Conference Record of the Thirty-Second Asilomar Conference on Signals, Systems & Computers. 1998; 2: 1251–1255.

64. Welch BL. The generalization of “Student's” problem when several different population variances are involved. Biometrika. 1947; 34.1/2: 28–35.

65. Student. The probable error of a mean. Biometrica. 1908; 6.1: 1-25.

66. Brown MB. A method for combining non-independent, one-sided tests of significance. Biometrics 1975; 31.4: 987-992.

67. Kost JT, McDermott MP. Combining dependent P-values. Stat Probab Lett. 2002; 60: 183–190.

68. Beit-Arieh I. Horvat ‘Uza and Horvat Radum: Two Fortresses in the Biblical Negev (Tel Aviv University Monograph Series 25). Tel Aviv University; 2007.

69. Na’aman N. A sapiential composition from Horvat ‘Uza. Hebrew Bible and Ancient Israel. 2013; 2: 221–233.

70. Beit-Arieh I, Freud L. Tel Malhata: A Central City in the Biblical Negev (Tel Aviv University Monograph Series 32). Tel Aviv University; 2015.

71. Aharoni Y, editor. Beer-Sheba I: Excavations at Tel Beer-Sheba, 1969-1971 Seasons. Tel Aviv University; 1973. pp. 71-75.

72. Beit-Arieh I. Tel ‘Ira. Israel Exploration Journal. 1981; 31: 224.

73. Beit-Arieh I. A First Temple period census document. Palest Explor Q. 1983; 115: 105-108.

74. Lemaire A. Notes d'épigraphie nord-ouest sémitique. Semítica. 1980; 30: 19-20.

75. Vritz V. Die ostraka. In: Fritz V, Kempinski A, editors. Khirbet el-Mšāš. Wiesbaden; 1983. pp. 133–137.

76. Cohen R. Inscriptions. In: Cohen R, Bernick-Greenberg H, editors. Excavations at Kadesh Barnea (Tell le-Qudeirat) 1976-1982 (IAA Reports 34). Jerusalem; 2007. pp. 245-254.

77. Starkey JL. Lachish I (Tell ed Duweir): Lachish Letters. Oxford University Press; 1938.

78. Lemaire A. Les écoles et la formation de la Bible dans l’ancien Israël (OBO 39). Fribourg and Göttingen; 1981.

79. Rollston CA. The Script of Hebrew Ostraca of the Iron Age: 8th-6th Centuries BCE. PhD Thesis, Johns Hopkins University. 1999.

80. Rollston CA. Scribal education in ancient Israel: The Old Hebrew epigraphic evidence. Bull Am Schools Orient Res. 2006; 344: 47-74.

81. Demsky A. Literacy in Ancient Israel. Jerusalem: Bialik; 2012 [Hebrew].

82. Na'aman N. Literacy in the Negev in the Late Monarchical Period. In Schmidt BB, editor. Contextualizing Israel's Sacred Writings. Ancient Literacy, Orality, and Literary Production (Ancient Israel and Its Literature 22). Atlanta; 2015. pp 47-70.

83. Faigenbaum-Golovin S, Shaus A, Sober B, Turkel E, Piasetzky E, Finkelstein I, Algorithmic handwriting analysis of the Samaria inscriptions illuminates bureaucratic apparatus in biblical Israel, PLOS ONE. 2019; 15.1: e0227452. https://doi.org/10.1371/journal.pone.0227452

84. Römer T. The So-Called Deuteronomistic History: A Sociological, Historical and Literary Introduction. London: T. & T. Clark; 2005.

85. Na'aman N. The Past that Shapes the Present: The Creation of Biblical Historiography in the Late First Temple Period and After the Downfall. Jerusalem: Yeriot; 2002 [Hebrew].

86. Finkelstein I. Jerusalem and Judah 600-200 BCE: Implications for Understanding Pentateuchal Texts. In: Dubovsky P, Markl D, Sonnet JP, editors. The Fall of Jerusalem and the Rise of the Torah. Tübingen; 2016. pp. 3-18.
